# A Fish-Focused Menu: An Interdisciplinary Reconstruction of Ancestral Tsleil-Waututh Diets

**DOI:** 10.1177/02780771241261235

**Published:** 2024-08-02

**Authors:** Meaghan Efford, Santiago de la Puente, Micheal George, Michelle George, Alessandria Testani, Spencer Taft, Jesse Morin, Jay Hilsden, Jennifer Zhu, Pengpeng Chen, Lindsey Paskulin, Ginevra Toniello, Villy Christensen, Camilla Speller

**Affiliations:** 1 Institute for the Oceans and Fisheries, 8166 University of British Columbia, Vancouver, BC, Canada; 2 6273 Norwegian Institute for Water Research, Oslo, Norway; 3 356093 Tsleil-Waututh Nation, North Vancouver, BC, Canada; 4 Department of Anthropology, 8166 University of British Columbia, Vancouver, BC, Canada

**Keywords:** zooarcheology, traditional diets, coast salish, indigenous-led research, interdisciplinary

## Abstract

The study of past subsistence offers archeologists a lens through which we can understand relationships between people and their homelands. səl̓ilwətaɬ (Tsleil-Waututh) is a Coast Salish Nation whose traditional and unceded territory centers on səl̓ilwət (Tsleil-Wat, Burrard Inlet, British Columbia, Canada). səl̓ilwətaɬ people were fish specialists whose traditional diet focused primarily on marine and tidal protein sources. In this research, we draw on the archeological record, ecology, historical and archival records, and səl̓ilwətaɬ oral histories and community knowledge to build an estimated precontact diet that ancestral səl̓ilwətaɬ people obtained from səl̓ilwət. Based on prior archeological research, we assume a high protein diet that is primarily (90–100 percent) from marine and tidal sources. The four pillars of səl̓ilwətaɬ precontact diets (salmon, forage fish, shellfish, and marine birds) offer anchor points that ensure the diet is realistic, evidence-based, and representative of community knowledge. We consider the caloric needs of adults, children, elders, and those who are pregnant or lactating. Finally, we consider the variation in the edible yield from different animal species and their relationships in the food web. Together, these data and anchor points build an estimated precontact diet averaged across seasons, ages, and biological sex from approximately 1000 CE up until early European contact in approximately 1792 CE. The reconstruction of səl̓ilwətaɬ lifeways and subsistence practices, which were based on a myriad of stewardship techniques, aid our understanding of the precontact səl̓ilwətaɬ diet and the relationship between səl̓ilwətaɬ and their territory.

## Introduction

**Figure 1.**
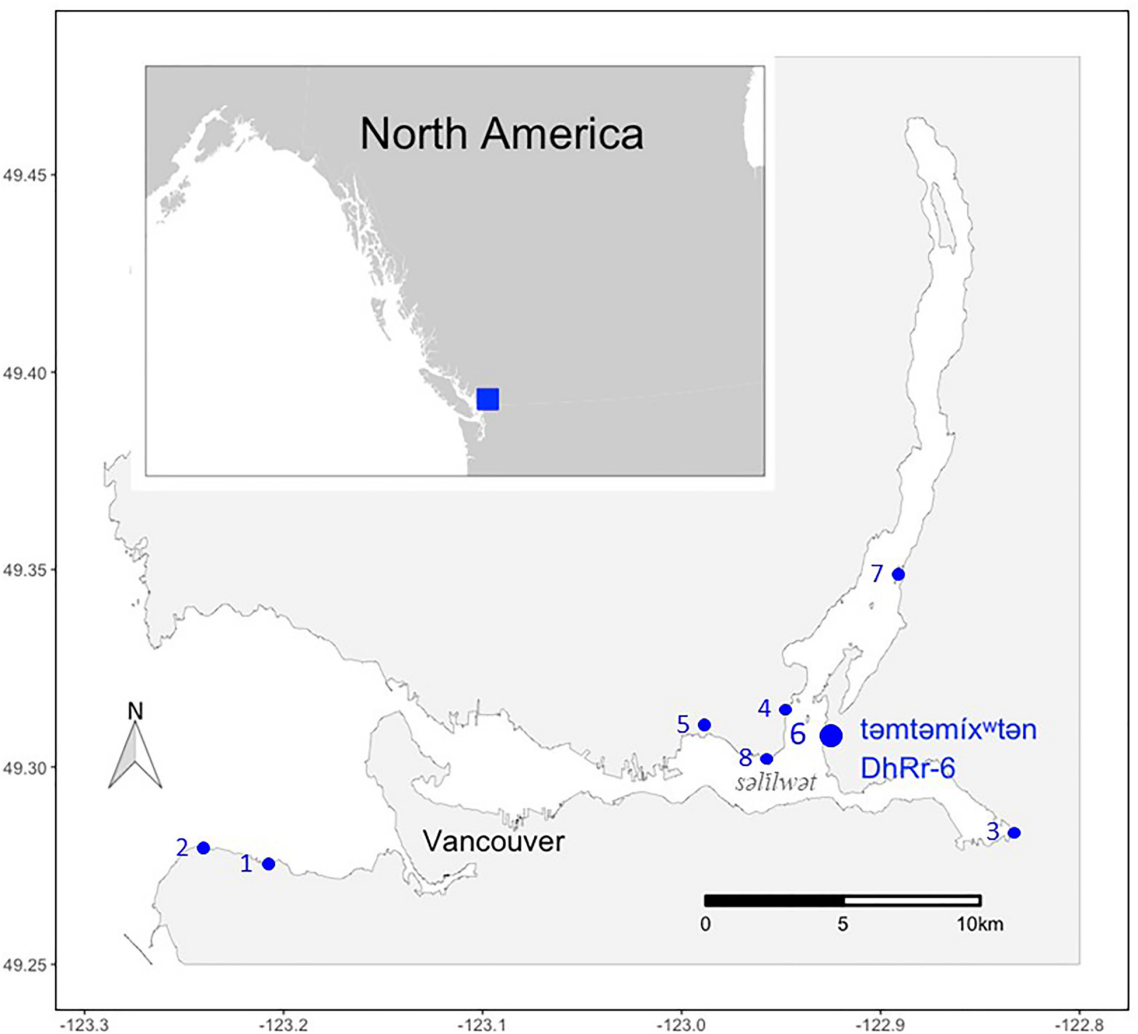
Map of səl̓ilwət (Burrard Inlet) with the following archeological sites: (1) qʷəɁápəɫp (DhRt-6, Locarno Beach), (2) Point Gray (DhRt-5), (3) seymamət (DhRq-1, Say-mah-mit, Noon's Creek), (4) Say-umiton (DhRr-18, Cove Cliff, Strathcona Park), (5) sʔəθnəc (DhRr-15 and 20, S’athnatch, IR No. 3), (6) təmtəmíxʷtən (DhRr-6, Tum-tumay-whueton, Belcarra Park), (7) Twin Islands (DiRr-16), (8) Whey-ah-wichen (DhRr-8, Cates Park). Created by V. Christensen and M. Efford 2023.

Archeological records can provide ample evidence of past subsistence and paint a more full picture of past ecological conditions that is not accessible by looking at fisheries data alone (Trost 2005; Lepofsky, Trost and Morin 2007; Cannon and Moss 2011; Pierson 2011). For səl̓ilwətaɬ (Tsleil-Waututh), food resources are integrated in stewardship ethics and provide connections between səl̓ilwətaɬ peoples and their territory. səl̓ilwətaɬ are a Coast Salish First Nation whose traditional and unceded territory is located in what is now known as North Vancouver, British Columbia, Canada (Figure 1). səl̓ilwətaɬ territory spans marine, freshwater, tidal, forest, and mountainous ecosystems. At the heart of the territory is Burrard Inlet, called səl̓ilwət in hən̓q̓əmin̓əm̓ (Tsleil-Wat, or a single səl̓ilwətaɬ person, in the downriver dialect of the Halkomelem language). səl̓ilwət and its watershed once supported the səl̓ilwətaɬ community (Morin 2014, 2015; Morin et al. 2018; Morin and Evans 2022) with salmon, forage fish, marine birds, and clams forming the core of the diet (Tsleil-Waututh Nation, 2016).

Calls for transdisciplinary approaches to efforts to reconstruct the past to better understand change over time and the state of our present-day environments have echoed through archeology, ecology, geography, and historical ecology (Butzer 1982a, 1982b; Swetnam, Allen and Betancourt 1999; Balée 2006; Briggs et al. 2006; Szabó 2010; Armstrong et al. 2017; Beller et al. 2017; Greer et al. 2018; Crabtree, Dunne and Wood 2021; Crumley, Bazan and Castrorao Barba 2021; Rick 2023). This research is an example of a transdisciplinary approach to dietary reconstruction as a collaborative research program led by səl̓ilwətaɬ. Archeology is by nature interdisciplinary, drawing on a wide range of disciplinary toolboxes including cultural anthropology, art history, ecology, taphonomy, geography, history, and more (Butzer 1982b; Reitz and Wing 2008; Moss 2011; Maggio 2018; Crumley, Bazan and Castrorao Barba 2021). However, it still relies on material remains that decompose or are otherwise impacted over time, and so combining archeological research into a transdisciplinary approach that draws on other sources of data and knowledge is beneficial and can aid in a better understanding of past lifeways and subsistence.

We investigate precontact səl̓ilwətaɬ connections to and relationships with the lands and waters of their ancestral territory through the reconstruction of səl̓ilwətaɬ precontact diet. We integrate archeology, ecology, historical and archival records, and səl̓ilwətaɬ traditional ecological and cultural knowledge to reconstruct a best estimate of the precontact diet of səl̓ilwətaɬ communities. Multidisciplinary research provides a better understanding of human-to-environment relationships over deep time compared to single-disciplinary approaches (Armstrong et al. 2017; Balée 2006; Beller et al. 2017; Crumley, Bazan and Castrorao Barba 2021). Our research is part of ongoing research on səl̓ilwətaɬ history and the relationships between ancestral and contemporary səl̓ilwətaɬ communities and their territory (Trost 2005; Lepofsky, Trost and Morin 2007; Pierson 2011; Morin and Hunt 2014; Morin 2015; Morin et al. 2018) and on səl̓ilwətaɬ subsistence and reconstruction of precontact ecology in Burrard Inlet (Morin et al. 2021; Morin et al. 2021; Taft et al. 2022; Efford et al. 2023; Morin, Evans and Efford 2023).

Through this research, we focus on reconstructing what marine food resources səl̓ilwət provided to sustain the ancestral səl̓ilwətaɬ community, and we assume that import of food to the səl̓ilwətaɬ community would be balanced by the export of foods out of səl̓ilwətaɬ territory. The available archeological evidence spans over 3,000 years BP, specifically at atqʷəɁápəɫp (Locarno Beach, DhRt-6), sʔəθnəc (S’athnatch, IR No. 3, DhRr-15 and 20), and təmtəmíxʷtən (“Tum-tumay-whueton,” Belcarra Park, DhRr-6) (Matthews 1955; Bouchard and Turner 1976; D. Rozen 1979; Lepofsky, Trost and Morin 2007; Morin 2014, 2015; Morin et al. 2018; Morin et al. 2021; Morin et al. 2021). However, our reconstruction of səl̓ilwətaɬ diet is based in the time period from approximately 1000 CE to European contact in approximately 1792 CE (Lamb 1984). This is to narrow our focus and limit the reconstruction to a more specific time period that includes more of the archeological sites along the shores of səl̓ilwət. Although exchange of food is an important component of Coast Salish kinship protocols and sustenance (H. G. Barnett 1955; Suttles 1960) it is beyond the scope of this study, which focuses solely on what səl̓ilwət could provide and does not consider imported or exported food. This research adds to archeological understandings within the broader perspective of Coast Salish Peoples (Moss 1993, 2011, 2016; Cannon and Moss 2011; Caldwell et al. 2012; McKechnie and Moss 2016).

### Study Area: səl̓ilwət

səl̓ilwət is located within the core of səl̓ilwətaɬ territory and remains central to the səl̓ilwətaɬ people to this day, with the contemporary home of the səl̓ilwətaɬ located along its shore (L. George 1991; Morin 2015; Tsleil-Waututh Nation, 2016; Morin et al. 2018, 2023; Tsleil-Waututh Nation's Treaty Lands and Resources Department, 2021; Morin and Evans 2022) (see Figure 1). səl̓ilwət and surrounding lands are currently also occupied by the Greater Vancouver region, home to 2.5 million people, and the Port of Vancouver, the largest port in Canada. The area is also home to a wide range of industrial, commercial, residential, and recreational activity. Before the colonization and urbanization of the area, however, səl̓ilwət was a healthy and productive ecosystem occupied by thousands of səl̓ilwətaɬ people with long-term stability in resource abundance (Pierson 2011; Morin 2015; Tsleil-Waututh Nation, 2016; Morin et al. 2021; Morin and Evans 2022; Efford et al. 2023; Morin, Evans and Efford 2023).

Four groups of marine foods have been especially important in traditional səl̓ilwətaɬ diets: Salmonidae spp., forage fish (including herring, smelt, anchovy and eulachon), shellfish, and marine birds (Tsleil-Waututh Nation, 2016). Thriving populations of marine fish living in səl̓ilwət precontact include Pacific salmon (*Oncorhynchus* spp.), Pacific herring (*Clupea pallasii*), eulachon (*Thaleichthys pacificus*), surf smelt (*Hypomesus pretiosus*), anchovy (*Engraulis mordax*), flatfish (various including *Hippoglossus stenolepis)*, and sturgeon (*Acipenser* spp.) (Morin and Evans 2022, p. 48). Pacific salmon are cultural and ecological keystone species in the Pacific Northwest (Garabaldi and Turner 2004; Moss 2016) and have been important components in Coast Salish diets and culture for millennia (Yang, Cannon and Saunders 2004; Butler 2008; Reid 2020; Atlas et al. 2021; Morin et al. 2021; Reid et al. 2022; Efford et al. 2023). The səl̓ilwətaɬ community harvested chum salmon (*O. keta*) in greater frequencies than other salmon species in səl̓ilwət (Morin et al. 2021; Morin et al. 2021; Efford et al. 2023). Chum appears to be the most abundantly available salmon species in səl̓ilwət, along with coho salmon (*O. kisutch*) and pink salmon (*O. gorbuscha*) (Hancock and Marshall 1986; Efford et al. 2023). Herring and their roe have been another staple of səl̓ilwətaɬ diets (Cannon 2000; Trost 2005; Pierson 2011; Morin 2015, p. 358,393,415; Moss 2016). The səl̓ilwət herring population, along with surf smelt and eulachon, suffered immense damage from 1880 to 1930 CE due to destructive and poorly managed colonial fishing practices, urban development, habitat destruction, and pollution (Morin, Evans, and Efford 2023). The herring population was extirpated from the eastern portion of səl̓ilwət in the 1880s (Morin, Evans and Efford 2023).

Marine birds are abundant in the archeological assemblages at təmtəmíxʷtən (DhRr-6), Twin Islands (DiRr-16), Say-umiton (DhRr-18) and seymamət (DhRq-1) (Morin 2015; Trost 2005; Pierson 2011) and səl̓ilwətaɬ traditional use studies (TUSs) tell us that ducks and other waterfowl in particular were especially abundant in the ecosystem (Morin and Evans 2022). səl̓ilwətaɬ communities hunted and trapped marine bird extensively (Trost 2005; Pierson 2011; Morin 2015; Morin and Evans 2022). Various dabbling ducks (*Anas* spp.) are particularly abundant (Trost 2005; Pierson 2011). Birds were abundant during the winter months when other food sources were less available, and were hunted with a variety of methods, including traps and nets (Morin 2015).

Shellfish including butter clams (*Saxidomus gigantea*), littleneck clams (*Leukoma staminea*), and cockles (*Clinocardium nuttallii*) have been a pillar of Coast Salish diets for millennia (H. G. Barnett 1938; Suttles 1960; R. L. Carlson 1996; Lepofsky, Trost and Morin 2007, 2015, 2021; Lepofsky and Caldwell 2013; Toniello et al. 2019). Archeological evidence shows these species have been part of səl̓ilwətaɬ diets for at least 3,000 years (Charlton 1972, 1977; Trost 2005; Lepofsky, Trost and Morin 2007; Pierson 2011; Lepofsky and Caldwell 2013; Morin 2014, 2015). The urban and industrial development within the Greater Vancouver area has caused immense shoreline damage, with a decrease of 945 hectares (55 percent) of tidal zone within səl̓ilwət from 1792 to 2022 (Taft et al. 2022, p. 18). The loss of so much of the tidal ecosystem represents a loss in shellfish habitat. Further, tidal zones also provide essential habitat to forage fish and salmon for whom this area is important habitat, and to marine birds who rely on shellfish and forage fish for food—an example of a cascading effect through the ecosystem (Pierson 2011; Taft et al. 2022, p. 7). Together, salmon, forage fish, shellfish, and marine birds form the foundation of our dietary reconstruction.

### səl̓ilwətaɬ Archeology and Historical Ecology

Coast Salish management of marine, tidal, and terrestrial environments has been explored through archeology and historical ecology research (Caldwell et al. 2012; Lepofsky and Caldwell 2013; Toniello et al. 2019, 2023). Indigenous resource management demonstrates that communities not only relied on animal and plant communities for sustenance, but part of the cultural relationship between people, place, and animals required active stewardship of those resources. səl̓ilwətaɬ relationships to the land and waters of their traditional territory is characterized by local ecological knowledge passed down through generations. This is evident in the words of former Elected səl̓ilwətaɬ Chief Leonard George:

When your first father was a – was just a child-man, that he used to roam throughout the inlet and he learned from all of the animals in the environment around him. He learned from the salmon the cycle of life and the highways of the ocean and why they would go out and the times they did and why they would return. He learned from the bird when the berries were ripe on the top of the mountain (L. George 1997).

səl̓ilwətaɬ communities are fish specialists and focused heavily on the marine and tidal environments for sustenance (Morin 2015; Morin et al. 2018; Morin and Evans 2022). The term hunter-gatherer (or even hunter-gatherer-fisher) does not fully encompass the intentional resource management and fish specialization that is apparent in səl̓ilwətaɬ history and archeology. Rather than opportunistic hunting and harvesting, there is a clear pattern of səl̓ilwətaɬ resource and land- and waterscape management and stewardship (Morin 2015; Morin and Evans 2022; Efford et al. 2023). Ancestral səl̓ilwətaɬ communities sustainably managed fisheries that specialized in salmon and forage fish using management techniques including sex-selective fishing (Efford et al. 2023; Morin, Evans and Efford 2023). Acknowledging this allows us to reconstruct an estimated precontact diet, knowing that the diet itself is tied to place and the animals who share it with humans.

## Methods

We use an interdisciplinary approach to precontact dietary reconstruction that draws on multiple data sources from archeology, ecology, historical records, and traditional ecological knowledge (TEK) from səl̓ilwətaɬ experts. We use this approach to mitigate disciplinary biases and to provide a best-estimate of what the precontact səl̓ilwətaɬ diet could have been, averaged across seasons. The zooarcheological record and səl̓ilwətaɬ traditional knowledge demonstrate that salmon, forage fish, marine birds, and clams form the heart of the precontact diet (Trost 2005; Pierson 2011; Morin 2015; Tsleil-Waututh Nation, 2016; Morin et al. 2018, 2023; Morin et al. 2021; Morin et al. 2021; Efford et al. 2023; Efford et al. 2023). Hence, we used these four food groups as anchor points in the process and assumed that they made up the most significant portions of the precontact səl̓ilwətaɬ diet compared to all other foods. The local ecology of the Inlet watershed provides a list of available foods, which is then compared to the archeological record. The nutritional needs of the population, including age, sex, and activity level are included, as is the edible yield of each food item (i.e., the portion of the animal or plant that is edible after processing). Seasonal variation in diet would have been pronounced: for example, chum salmon is available in the fall, whereas herring and eulachon are available in the spring (Suttles 1987; Duffield and McHalsie 2001; McKechnie et al. 2014; Morin 2015). Seasonally specific occupation sites offered local access to seasonally available resources (Kelly 1983; Morin 2015). However, in this research we use an annual average across all seasons. These components together make up the reconstructed traditional səl̓ilwətaɬ diet.

### Dietary Reconstruction

Our process for reconstructing traditional diets considered archeology, historical ecology, nutritional guidelines, and səl̓ilwətaɬ traditional ecological and cultural knowledge data. Including səl̓ilwətaɬ traditional ecological and cultural knowledge increases the accuracy of the diet reconstruction that would not be possible by looking at any of these datasets alone. We use three main components to develop the diet, which also provides limitations:

1. local ecology and zooarcheological evidence, which provide a potential diet;
2. the nutritional needs of an active population; and
3. səl̓ilwətaɬ traditional dietary focus, which identify which foods are preferred as main contributors to the daily diet versus those that contribute less.

Limitations (chosen boundaries or parameters) guide and structure the process by ensuring the reconstruction is realistic. For example, the local ecology limitation ensures we only include animals and plants that could be present given the local ecology. The nutritional needs limitation ensures the diet is healthy, i.e., not exceeding the daily safe protein amount. Dietary focus and cultural norms limitation offers which foods are preferred as main contributors to the daily diet versus those that contribute less. We know that the precontact səl̓ilwətaɬ diet is high in protein based on səl̓ilwətaɬ traditional ecological and cultural data. In our diet reconstruction, we inputted a daily protein serving of under 300 g in order to further avoid any possible protein poisoning within our reconstruction (Speth et al. 1991, p. 106). As the diet is built with a primary focus on protein, carbohydrate-rich foods, like plant foods, are not highlighted, but this does not mean that they were not important and consistent contributors to the diet. As they would have provided less protein, fat, and calories per serving, plants are less emphasized in this analysis.

Our initial draft diet resulted in an average of 43 percent of calories coming from protein, meaning that the diet requires a minimum of 921 calories from protein. Drawing on all data sources to create a list of possible foods and food groups, we presented draft iterations of the diet to səl̓ilwətaɬ knowledge holders and coauthors. Based on their feedback, including adjustments to archeologically less visible or invisible animals like sturgeon, crab, and plant foods, we refined the diet. The iterative approach implemented allowed us to account for taphonomic factors, which will differentially impact archeological fish and animal remains based on their fragility and robustness (Bartosiewicz 2008; Reitz and Wing 2008; Gifford-Gonzalez 2018). Finally, we based the relative contribution of different salmon species on previously published work (Morin et al. 2021; Morin et al. 2021; Efford et al. 2023).

We calculated the dietary composition of most groups using a database of food composition, with some groups comprised of several foods combined (e.g., “berries,” “root vegetables,” and “marine white fishes”) (M. Smith 2018). The groups that are not included in the 2018 database required different data sources. Some examples of these include sea lions (Arnold et al. 2006, p. 42), seals, eulachon, herring spawn (Moss 2016, p. 650), and spiny dogfish (I. Smith 2011, p. 12). On average, each gram of protein provides four calories, and each gram of fat provides nine calories (National Agricultural Library U.S. Department of Agriculture, 2023). These limitations help ensure that the diet is reasonable and safe to consume. The daily serving of each food is averaged across a yearly harvest: we do not assume that all these foods would be eaten daily, rather this is the average daily amount of each food from the annual harvest.

We assigned each harvested food group (*N* = 33) their calories, protein, and fat per 100 g. We drafted an estimated daily serving size per person based on protein in grams to divide the foods into a daily “menu.” We used protein as the focus due to the significance of protein in səl̓ilwətaɬ traditional ecological and cultural data. Once we determined the daily food menu we then extrapolated to the yearly harvest by multiplying the daily amount by 365 (averaged across seasons).

### səl̓ilwətaɬ Traditional Ecological and Cultural Knowledge

Incorporation of səl̓ilwətaɬ traditional ecological and cultural knowledge is a key component of this study. We accessed səl̓ilwətaɬ TEK from səl̓ilwətaɬ TUS projects (TWN, 1998, 2000, 2011; Morin and Evans 2022; Taft et al. 2022; Efford et al. 2023) and through direct collaboration with səl̓ilwətaɬ knowledge holders. Micheal George, Michelle George, Gabriel George, and Carleen Thomas provided ecological and cultural knowledge of their Ancestor's diets, which was used to fill in gaps in the other data sources and ensured the estimated diet was realistic and in accordance with səl̓ilwətaɬ oral histories and science.

Frequent collaborative review meetings with səl̓ilwətaɬ knowledge holders provided research guidance in addition to the TEK. We chose collaborative review meetings rather than ethnographic interviews due to the preference for səl̓ilwətaɬ contributors, and to include səl̓ilwətaɬ collaborators as co-creators of the research rather than interviewees. During initial review meetings, research questions were developed together to address specific questions of interest to səl̓ilwətaɬ. Draft research questions based on available data were presented for refinement. In subsequent meetings, methods and results were presented to səl̓ilwətaɬ knowledge holders and staff for consideration and assessment. Questions were posed to səl̓ilwətaɬ regarding their perspective on: (1) how səl̓ilwətaɬ is portrayed or discussed in the research; (2) the implications of this research for səl̓ilwətaɬ; and (3) next steps, including any questions the research leaves unanswered for səl̓ilwətaɬ. Finally, in editing the iterations of the reconstructed diet, səl̓ilwətaɬ collaborators aided in assessing the relative dietary contribution of each food group.

We held review meetings frequently over the research process, ensuring səl̓ilwətaɬ access and opportunity for assessment and questions. səl̓ilwətaɬ collaborators offered səl̓ilwətaɬ science and TEK to fill in the gaps that the archeology and historical/archival records could not address. Additionally, these review meetings established how taxa were grouped based on how they would have been considered as part of the precontact səl̓ilwətaɬ diet. This was an important component of this research as the research falls under səl̓ilwətaɬ data sovereignty and leadership. səl̓ilwətaɬ knowledge of past subsistence practices tells us that səl̓ilwətaɬ communities harvested across the ecosystem, with salmon, seals, ducks, and general references to fish occurring most frequently (Matthews 1955; Wells 1966; Carter 1972; Bouchard and Kennedy 1986; P. George 1990).

### Historical and Archival Sources

We performed a review of historical and archival records that pertain to our study area and the surrounding environment. We sought to gain an understanding of the most commonly reported taxa and to establish whether there were any taxa mentioned that were not visible in the archeological record. Historical and archival references to food resources in the səl̓ilwət watershed area include a wide variety of mammals, fish, shellfish, and birds (Vancouver 1798, 1984; Simpson 1847; Menzies and Forsyth 1923; Wagner 1933; H. Barnett 1935; Carter 1966; Wells 1966, 1987; Johnson 1971; Bouchard and Kennedy 1976; Harris 1978; D. L. Rozen 1985; P. George 1990; L. George 1997; MacDonald et al. 1998; Matthews 2011; Morin 2015; Barman 2016). In the review of the historical and archival records, it became clear that the most commonly mentioned taxa include seals, salmon, sturgeon, herring, flatfish, surf smelt, eulachon, ducks, crabs, and clams or other bivalve mollusks (Vancouver 1798, 1984; Simpson 1847; Menzies and Forsyth 1923; Wagner 1933; H. Barnett 1935; Matthews 1955, 2011; Carter 1966; Wells 1966, 1987; Johnson 1971; Harris 1978; D. L. Rozen 1985; P. George 1990; L. George 1997; MacDonald et al. 1998; Morin 2015; Barman 2016). Salmon is reported extensively as an important resource and part of the local ecology (Simpson 1847; Wagner 1933; Matthews 1955; Carter 1972; Harris 1978; D. L. Rozen 1985; L. George 1997; MacDonald et al. 1998; Barman 2016), as are herring (Simpson 1847; Matthews 1955; P. George 1990), surf smelt (Matthews 1955, 2011), and ducks (Matthews 1955, 2011).

The majority of these records discuss the period of 1791–1858 CE, with few focusing on the period before Europeans started to settle in the area. These records provide valuable information that can be evaluated in tandem with the archeological record, local ecological knowledge, and the current ecology of the study area. Despite these records having been written after contact, they predate the period of time during which the urbanization and settlement of the area began in earnest in the 1880s.

### Archeological Data - Fauna

Archeological evidence from səl̓ilwət offers insight into the local ecology and availability of foods from before European contact (Coupland 1991; Pierson 2011; Morin 2014, 2015; Morin and Hunt 2014; Morin et al. 2018; Morin et al. 2021; Morin et al. 2021; Guttmann 2022; Morin and Evans 2022; Efford et al. 2023). We performed a review of reported faunal material (animal remains) from the following archeological sites, which provides evidence of those species were part of precontact diet (Lepofsky, Trost and Morin 2007; Pierson 2011; Morin 2015; Morin et al. 2021; Morin et al. 2021; Morin, Evans and Efford 2023) (Figure 1, Table 1). We then conducted a zooarcheological inventory of faunal material collected from təmtəmíxʷtən (DhRr-6) (see Figure 1 and Table 1) by Arthur Charlton in the 1970s (Charlton 1972, 1977, 1980). We focused on two excavation units, Unit 116–118 N, 0–2 W and Unit 116–118 N, 4–6 W. Number of identified specimens was quantified for fish vertebrae, other fish elements (nonvertebrae), bird bones, and mammal bones using morphological identification (Grayson 1984; Mitchell 1990; Reitz and Wing 2008; Driver 2011; Gifford-Gonzalez 2018).

**Table 1.** Archeological Sites Along the Shores of səl̓ilwət (see Figure 1) included in zooarcheological assessment of possible contributing foods. New data analysis using aDNA and ZooMS performed on faunal material from təmtəmíxʷtən (DhRr-6).

#	Archeological Site	Borden #	Date range of occupation	Citations
1	qʷəɁápəɫp (Locarno Beach)	DhRt-6	3300–2400 cal BP	Rozen 1979:41; Bouchard and Turner 1976; Matthews 1955, Morin 2015
2	Point Gray	DhRt-5	2000–1500 cal BP	Coupland 1991, Morin 2015
3	seymamət (Say-mah-mit, Noon's Creek)	DhRq-1	2000–1501 cal BP to 1001 cal BP	Morin 2015, Morin et al. 2018, Morin et al. 2021, Morin et al. 2021, Pierson 2011
4	Say-umiton (Cove Cliff, Strathcona Park)	DhRr-18	1500–1001 cal BP to 500–100 cal BP	Lepofsky, Trost and Morin 2007, Morin 2015, Morin et al. 2018, Morin et al. 2021, Morin et al. 2021, Trost 2005
5	sʔəθnəc (S’athnatch, IR No. 3)	DhRr-15 and 20	≥3500 cal BP to 500–100 cal BP	Lepofsky, Trost and Morin 2007, Morin 2015, Morin et al. 2018
6	təmtəmíxʷtən (‘Tum-tumay-whueton’, Belcarra Park)	DhRr-6	3500–3001 cal BP to 500–100 cal BP	Efford et al. 2023, Lepofsky, Trost and Morin 2007, Morin 2014, 2015, Morin et al. 2018, Morin et al. 2021, Morin et al. 2021, Pierson 2011
7	Twin Islands	DiRr-16	-	Pierson 2011
8	Whey-ah-wichen (Cates Park)	DhRr-8	2500–2100 cal BP to 500–100 cal BP	Lepofsky et al. 2007, Morin 2015, Morin et al. 2018

^a^DNA = ancient DNA; ZooMS = Zooarcheology by mass spectrometry.

Across all sites, salmon, herring, northern anchovy, and spiny dogfish vertebrae were found in large quantities, particularly the salmon vertebrae. Black-tailed deer (*Odocoileus hemionus*) bones, which are robust and preserve very well archeologically, are also found in abundance. Although we did not quantify bivalves in this study, clam shells are abundant in the təmtəmíxʷtən archeological record, numbering many millions of total shells at the site. Based on this, there is a wide variety of foods from across the ecosystem that we include in the dietary reconstruction, providing seasonal variety.

To obtain greater resolution on past səl̓ilwətaɬ diets, we conducted new biomolecular analyses on faunal material from təmtəmíxʷtən (DhRr-6), including Zooarcheology by mass spectrometry (ZooMS) and ancient DNA (aDNA) analysis to taxonomically identify a subset of the zooarcheological assemblage. For the remaining sites, we reviewed faunal data within archeological reports. All eight archeological sites are included because they have existing or new archeological evidence of faunal remains upon which we can base our dietary reconstruction. We do not calculate marine species abundance in this study (see Lepofsky, Trost and Morin 2007).

ZooMS, or collagen peptide fingerprinting, is a rapid and cost-effective method of taxonomic identification (Collins et al. 2010; Richter et al. 2022; Efford et al. 2023). We conducted both ZooMS and aDNA analysis on archeological faunal samples recovered from təmtəmíxʷtən (DhRr-6) at the AdαPT Lab at the University of British Columbia (see Supplemental Information for full details on the laboratory methods and results). ZooMS analysis was conducted on fifty-two zooarcheological remains from təmtəmíxʷtən (see Supplemental Table 1), forming a complement to previously published ZooMS analysis of 230 salmon bones (Efford et al. 2023). In addition to the ZooMS analysis, aDNA analysis was conducted on seventeen of the samples to clarify or refine identifications (see Supplemental Table 2). All samples were chosen using a randomization method, using a random number generator to choose from a sample pool, established as all potential samples from an individual excavated level. See Supplemental Table 1 for details on chosen samples. We do not include paleobotanical data in this reconstruction as the focus of our research is on animal protein, and there is no previously published paleobotanical data for our study area.

### Nutritional Needs

To estimate the nutritional needs of the precontact səl̓ilwətaɬ communities, we first examine stable-carbon isotopes of Coast Salish Ancestral human remains, the known caloric intake of other hunter-gatherer populations, and use the US Department of Agriculture/US Department of Health and Human Services Dietary Guidelines for Americans, 2020–2025 (see Table 2). Stable-carbon isotope analyses of səl̓ilwətaɬ Ancestral Remains from təmtəmíxʷtən demonstrates that the individual (who lived approximately 2190 ± 90 cal. BP) relied heavily on marine and tidal sources of food (96 percent), with relatively little coming from their terrestrial environment (Chisholm, Nelson and Schwarcz 1983; Schwarcz, Chisholm and Burchell 2014). We used a weighted average of the caloric needs across all groups and accommodate for the differing proportion of the population each group represents. In this reconstruction, children represent 30 percent of the population, and the average caloric needs for their group across ages 2–18 are 1776 (see Table 2). We recognize that this is a wide range in ages, developmental stages, and nutritional needs, and the age range itself is based on a contemporary adulthood age range, however refining the resolution of this group is not within the scope of this research. Including all categories, this comes out to 2120 total average calories per day for each individual across the population. We use the following equation to reach the average caloric needs per person:

$$\begin{array}{rl} & \mathrm{Weighted}\;\mathrm{average}\;\mathrm{of}\;\mathrm{Children}+\mathrm{Adults}+\mathrm{Pregnant}/\\ & \;\mathrm{lactating}\;\mathrm{Adults}+\mathrm{Elders}=2120\;(1776*0.30)\\ & \;+(2313*0.45)+(2406*0.10)+(2040*0.15)=2120 \end{array}$$

**Table 2.** Caloric Needs for a Moderately Active Population Based on the US Department of Agriculture/US Department of Health and Human Services Dietary Guidelines for Americans, 2020–2025 (U.S. Department of Agriculture & U.S. Department of Health and Human Services, 2020, pp. 112,140–141).

Category	Percentage of population	Age range	Range of required calories per person per day	Average required calories per person per day
Children	30	2–18	1000–2400	1776
Adults	45	19–55	2100–2500	2313
Adult men		19–55	2400–2800	2600
Adult women		19–55	1800–2200	2025
Pregnant or lactating adult*	10	19–55	2181–2581	2406
Elders	15	56+	2000–2100	2080
Elder women		56+	1800	1800
Elder men		56+	2200–2400	2280

* Pregnant or lactating Adult based on Adult Women amount plus 380.50.

The reported caloric needs of the Hadza of northern Tanzania, a traditional hunter-gatherer community, show similar numbers to the US Guidelines for adults, with adult Hadza women consuming 1,900 calories per day and adult Hadza men consuming 2,600 calories daily (Pontzer et al. 2012, 2016) (see Table 2 for the US Guidelines). While the Hadza have a very different culture and diet and live in a very different environment compared to səl̓ilwətaɬ precontact communities, they are one of the few communities in the world who continue to live as hunter-gatherers. Research has shown that their caloric needs and energy expenditure are not all that different than moderately active adults in Europe, despite living a more active life (Pontzer et al. 2012, 2016; Pontzer 2017). We chose to use the “moderately active” values from the American dietary guidelines, rather than “active” to represent an active fisher-hunter-gatherer population with long-term land- and waterscape management practices. For moderately active populations, these dietary guidelines recommend an average of 2,025 calories daily for adult women (2,406 if pregnant or lactating), 2,600 for adult men, 2,080 for adults over 55 years, and a wide range for children ages 2–18 from 1,000 to 2,400 averaging 1,776 (see Table 2) (U.S. Department of Agriculture & U.S. Department of Health and Human Services, 2020).

#### Avoiding “Protein Sickness” and “Rabbit Starvation”

Diets that rely on lean meats are not nutritionally whole, and can cause “rabbit starvation,” or protein poisoning, a phenomenon that describes cases where relying on lean meat sources results in people succumbing to malnutrition within weeks due to issues metabolizing the protein (Speth and Spielmann 1983; Bilsborough and Mann 2006; Kelly 2007; Tushingham, Barton and Bettinger 2021). The safe protein ceiling is approximately 300 g per day (Cordain et al. 2000; Mann 2000; Bilsborough and Mann 2006; Speth 2022, p. 52). Human bodies require fat and/or carbohydrates to properly metabolise protein and avoid overloading the liver (Bilsborough and Mann 2006, p. 133). In high-latitude hunter-gatherer communities, this can occur in the spring, when mammals that are hunted for food are themselves struggling to find enough food and are far less fatty than they would be during the summer and fall.

We look to traditional Inuit diets as an example of traditional diets that rely heavily on marine and tidal foods, with high levels protein and fat and very limited quantities of carbohydrates. Traditional Inuit diets rely on a wide variety of mammals, fish, shellfish, and birds and is heavy in protein, fats, Omega-3 fatty acids, vitamins, and minerals (Egeland et al. 2006; Fediuk 2000). The traditional Inuit diet indicates that it is possible for high protein, high-fat diets to be healthy and not risk heart or liver health. As the precontact səl̓ilwətaɬ diet is naturally very high in fat, with the protein primarily coming from marine and tidal foods and supplemented with red meat, we avoid concerns regarding balancing protein with either fat or carbohydrates.

### Edible Yield vs. Live Weight

The live weight of an animal is the average weight of the animal in life. The edible yield (or edible portion) is the average yield of each individual animal after the inedible portion is removed. For example, the average live weight of spiny dogfish (*Squalus acanthias*) is 4.9 kg, and the average edible yield is 36 percent (Crapo, Paust and Babbitt 1993, p. 5). This means that 64 percent of an average spiny dogfish is inedible, such as the bones and viscera. To translate a live harvest into a diet of edible foods, we use the edible yield for all foods, so 10 g of a given food is 10 g of edible food. Note that what is considered “edible” for any given food will vary from person to person, and from community to community, as well as within the context of food preparation. Our edible yield estimates are based on secondary data (Crapo, Paust and Babbitt 1993; Fediuk 2000; Ashley 2002; Arnold et al. 2006; I. Smith 2011; Moss 2016; M. Smith 2018) and do not consider the use of bones, fish heads, and oil. However, these “non-edible” portions can be processed and made to be edible. This adds a layer of uncertainty to our estimates, yet our edible yield calculations are likely conservative.

## Results

Across all data sets, the results demonstrate a widely diverse precontact səl̓ilwətaɬ diet, with a primary focus on fish and tidal foods, including clams and marine birds and terrestrial protein sources offering a small contribution. Our estimated diet reflects this variety while also reflecting the preference for the four primary food sources. Table 3 reports the food groups found in the ecosystem that we include in the dietary reconstruction. Note that Table 3 does not include all archeologically or ecologically represented species and groups, and only includes those that were used to reconstruct the protein, fat, and calories contributing to the dietary reconstruction.

**Table 3.** Plant and Animal Food Groups Found in the səl̓ilwət (Burrard Inlet) Ecosystem Used to Reconstruct the Diet, Based on Historical/Archival Resources and səl̓ilwətaɬ Traditional Ecological and Culture Knowledge, and Archeological Record(*) (see Lepofsky, Trost and Morin 2007, Morin 2015, Pierson 2011, and Trost 2005, and the faunal quantification and ancient DNA and Zooarcheology by Mass Spectrometry (ZooMS) analysis conducted for this research). This table does not represent all archeologically represented groups or species, and only represents what was used to establish the protein, fat, and calories included in the dietary reconstruction.

Group	Species
Ungulates	
	*Artiodactyla spp.
	*Black-tailed deer (*Odocoileus hemionus*)
	*Elk (*Cervus elaphus*)
Small terrestrial mammals	
	*Beaver (*Castor canadensis*)
	*Racoon (*Procyon lotor)*
	*Small canid (*Candiae*)
	*Snowshoe hare (*Lepus americanus*)
	*Squirrels (*Sciuridae* spp.)
Plant foods	
	Berries (various)
	Roots (various)
	Greens/medicinal plants (various)
Fungi	
	Wild mushrooms (various)
Birds	
	*Bay ducks (*Aythya* spp.)
	*Canada goose (*Branta canadensis*)
	*Ducks (*Anas* spp.)
	*Gull (*Laridae*)
	*Harlequin duck (*Historionicus historionicus*)
	*Horned grebe (*Podiceps auritus*)
	*Loons (*Gaviidae* spp.)
	*Surf scoter (*Melanitta perspicillata*)
	*Swan (*Cygnus* spp.)
Marine mammals	
	*Harbour seal (*Phoca vitulina*)
	Sea lions *(Zalophus wollebaeki)*
Marine white fishes	
	*Cod (*Gadidae*)
	Haddock (*Melanogrammus aeglefinus*)
	*Lingcod *(Ophiodon elongatus)*
	*Pacific cod *(Gadus macrocephalus)*
	Pollock (*Pollachius pollachius)*
	Whiting/hake *(Merlangius merlangus)*
Flatfish	
	*English sole (*Parophrys vetulus*)
	*Flathead sole (*Hippoglossoides elassodon*)
	*Flatfish spp. (Pleuronectiformes)
	*Pacific halibut *(Hippoglossus stenolepis)*
	*Rock sole (*Lepidopsetta bilineata*)
	*Sand sole (*Psettichthys melanostictus*)
	*Starry flounder (*Platichthys stallatus*)
Salmonidae	
	*Chinook salmon *(Oncorhynchus tshawytscha)*
	*Chum salmon *(Oncorhynchus keta)*
	*Coho salmon *(Oncorhynchus kisutch)*
	*Pink salmon *(Oncorhynchus gorbuscha)*
	*Sockeye salmon *(Oncorhynchus nerka)*
Other fishes	
	*Big skate (*Raja binoculata*)
	*Eulachon *(Thaleichthys pacificus)*
	*Northern anchovy *(Engraulis mordax)*
	*Northern sculpin (*Icelinus borealis*)
	*Pacific Herring *(Clupea pallasii)*
	*Perch (*Embiotocidae*)
	*Red irish lord (*Hemilepidotus cf hemolepodtus*)
	*Rockfish (*Sebastes* spp.)
	*Rock greenling (*Hexagrammos lagocephalus*)
	*Pacific staghorn sculpin (*Leptocottus marmoratus*)
	*Sculpin (*Cottidae* spp.)
	*Spiny dogfish *(Squalus acanthias)*
	*Stickleback (*Gasterosteidae* spp.)
	*Sturgeon (*Acipenser* spp.)
	*Surfperch (*Amphistichus rhodoterus*)
	*Surf smelt *(Hypomesus pretiosus)*
Crabs	
	*Dungeness Crab *(Metacarcinus magister)*
	*Redrock Crab *(Cancer productus)*
Bivalves	
	*Blue mussel (*Mylitus edulis*)
	*Butter clams *(Saxidomus gigantea)*
	*Clams
	*Cockles *(Clinocardium nuttalii)*
	*Horse clams *(Tresus nuttalii)*
	*Pacific littleneck clams (*Protothaca staminea*)
	*Macoma spp.
	*Native oyster (*Ostrea lurida*)
	*Pacific littleneck clam (*Protothaca staminea*)
	*Pacific oyster (*Crassostrea gigas*)
Echinoderms	
	*Urchins (*Echinoidea* and *Strongylocentrotus* spp.)
	Sea cucumbers (*Holothuroidea* spp.)
Other marine foods	
	Octopus (*Octopodidae spp.*)
	Shrimp (*Pandalus platyceros* and others)
	Squids (*Teuthoidea* spp.)

Of the ZooMS analysis conducted on fifty-two zooarcheological remains, fifteen samples were identified as deer (*Odocoileus sp.*), and three as either elk (*Cervus canadensis*) or moose (*Alces alces*), as these species cannot be distinguished using ZooMS (Von Holstein et al. 2014). Six of the samples were identified as seals (Phocidae), four as bovid/cervid, four as birds (including two Anatids [ducks, geese, swans]), two as musteloids, two as carnivores, one as bear, one as humpback whale (*Megaptera novaeangliae*), and one as canid (see Supplemental Table 1). Four non-salmon fish were also identified including two samples possibly representing starry flounder (*Platichthys stellatus*) and two as rockfish (*Sebastes* sp.); given that these samples were identified using ZooMS reference databases developed for Atlantic fish species (Buckley et al. 2022; Dierickx et al. 2022), their identifications are listed as probable.

For the aDNA analysis conducted on seventeen zooarcheological remains, analysis targeted multiple fragments of mitochondrial DNA (see Supplemental information for full details). One of the musteloids was identified as striped skunk (*Mephitis mephitis*), and ten of the bovid/cervids were identified as elk (*Cervus canadensis*, *n* = 5), mountain goat (*Oreamnos americanus*, *n* = 1), and black-tailed deer (*Odocoileus hemionus*, *n* = 4) (see Supplemental Table 2). The remaining musteloid, two of the carnivores, and three of the bovid/-cervids could not be identified to species.

The review of zooarcheological remains from təmtəmíxʷtən demonstrated a bias towards robust and taphonomically resistant faunal remains, including mammal bones, and fish vertebrae (see Table 4 and Table 5). Salmon vertebrae were by far the most abundant bone type, making up the majority of all fish vertebrae. Robust animal remains, such as clam shells and ungulate bones are overrepresented in the archeological record due to their hardiness, which offers resistance to taphonomic factors. Sturgeon, crabs, octopus, and sea cucumbers are all often archeologically invisible or underrepresented species that would have been underrepresented without səl̓ilwətaɬ cultural knowledge to balance the archeological analysis.

**Table 4.** Number of Identified Specimens (NISP) Quantities of Fish Vertebrae, Fish Bones (Nonvertebrae, Indicated with *), Bird Bones, and Mammal Bones, from Excavation Unit 116 to 118 N, 4–6 W. Includes Levels 1–9 and 11–18.

EU	Level (cm)	Context	Bag weight (g)	NISP: fish vertebrae	NISP: fish*	NISP: bird	NISP: mammal
116–118 N, 4–6 W	0–10	Level 1	47	29	10	1	21
116–118 N, 4–6 W	10–20	Level 2	77	33	3	0	20
116–118 N, 4–6 W	20–30	Level 3	142	35	0	6	53
116–118 N, 4–6 W	30–40	Level 4	261	85	6	9	53
116–118 N, 4–6 W	40–50	Level 5	318	280	20	16	68
116–118 N, 4–6 W	50–60	Level 6	268	93	0	12	89
116–118 N, 4–6 W	60–70	Level 7	444	27	72	12	101
116–118 N, 4–6 W	70–80	Level 8	612	218	37	8	173
116–118 N, 4–6 W	80–90	Level 9	497	197	25	18	110
116–118 N, 4–6 W	100–110	Level 11	208	350	43	15	30
116–118 N, 4–6 W	110–120	Level 12	271	401	31	15	35
116–118 N, 4–6 W	120–130	Level 13	369	323	67	21	64
116–118 N, 4–6 W	130–140	Level 14	440	421	112	13	75
116–118 N, 4–6 W	140–150	Level 15	360	200	182	27	118
116–118 N, 4–6 W	150–160	Level 16	876	46	45	11	69
116–118 N, 4–6 W	160–170	Level 17	120	135	120	6	35
116–118 N, 4–6 W	170–180	Level 18	83	8	37	2	27

**Table 5.** Number of Identified Specimens (NISP) Quantities of Fish Vertebrae, Fish Bones (Nonvertebrae, Indicated with *), Bird Bones, and Mammal Bones, from Excavation Unit 116–118 N, 0–2 W. Includes Levels 1–17.

EU	Level (cm)	Context	Bag weight (g)	NISP: fish vertebrae	NISP: fish*	NISP: bird	NISP: mammal
116–118 N, 0–2 W	0–10	Level 1	366	2	0	0	8
116–118 N, 0–2 W	10–20	Level 2	199	9	0	0	35
116–118 N, 0–2 W	20–30	Level 3	236	14	3	0	37
116–118 N, 0–2 W	30–40	Level 4	251	28	0	0	64
116–118 N, 0–2 W	40–50	Level 5	685	230	127	9	100
116–118 N, 0–2 W	50–60	Level 6	755	1,000+	21	5	66
116–118 N, 0–2 W	60–70	Level 7	1082	1,600+	64	9	68
116–118 N, 0–2 W	70–80	Level 8	1621	3,000+	287	12	61
116–118 N, 0–2 W	80–90	Level 9	297	220	24	6	44
116–118 N, 0–2 W	90–100	Level 10	162	54	16	4	28
116–118 N, 0–2 W	100–110	Level 11	155	51	7	1	30
116–118 N, 0–2 W	110–120	Level 12	166	22	2	4	34
116–118 N, 0–2 W	120–130	Level 13	379	220	18	2	62
116–118 N, 0–2 W	130–140	Level 14	156	7	12	3	25
116–118 N, 0–2 W	140–150	Level 15	300	114	2	2	30
116–118 N, 0–2 W	150–160	Level 16	458	45	50	10	4
116–118 N, 0–2 W	160–170	Level 17	47	2	0	0	5

**Figure 2.**
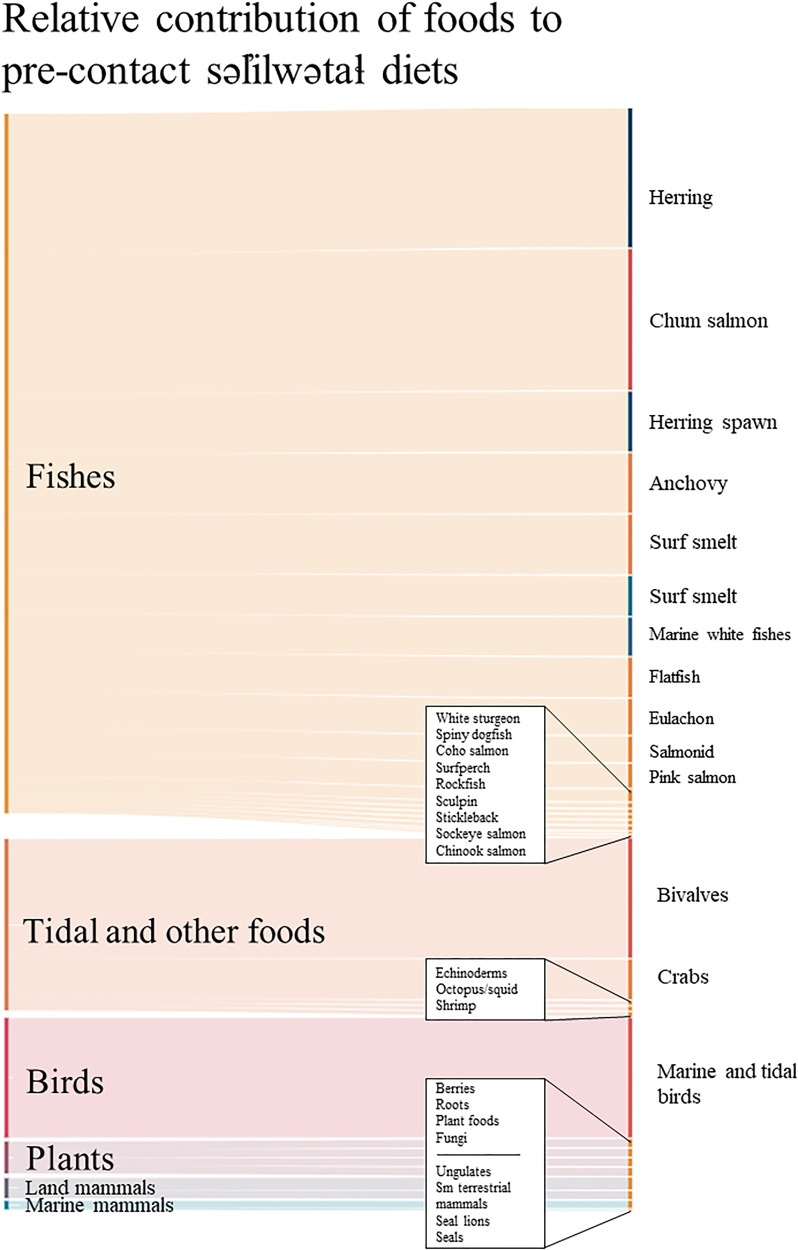
Diversity of the precontact səl̓ilwətaɬ diet organized by group. Figure shows the results of precontact səl̓ilwətaɬ dietary reconstruction. Created using Visual Paradigm, Efford 2024.

The resulting diet shows the wide variety of foods that would have been sustaining the ancestral səl̓ilwətaɬ community over many generations prior to European contact. The səl̓ilwətaɬ specialization in fish is clear, as is the focus on marine and tidal foods (see Figure 2 and Table 6). Figure 2 was created using the daily serving in grams for each food group reported in Table 6 and presents the relative contribution of each food group to the average daily diet. Table 6 includes the most significant collected data (For each Group: gProtein/100 g; Protein daily serving (g); gFat/100 g; Fat daily serving (g); Live weight in kg; edible percentage out of 1.00) used to generate new results (Daily serving (g); Percent of whole; Live amount per family of 4 per week). The diet reconstruction presented in Table 6 and Figure 2 represents an average across seasons, ages, and sex. The 1,312 g of food daily offers an average of 232 g of protein, 126 g of fat, and 2,242 calories (see Table 6) for a moderately active fisher-hunter-gatherer population relying on, specializing in, and managing marine and tidal foods, along with a wide variety of foods across their local ecosystem. The result is 41 percent of calories coming from protein, 51 percent of calories coming from fat, and 8 percent of calories coming from carbohydrates. This meets our required minimum and falls under the safe protein ceiling of 300 g per day. It exceeds the minimum required calories per person, which indicates that our reconstructed diet more than meets the needs of the population as it is modeled in this research. The result is 92 percent of the dietary protein coming from marine and tidal sources.

**Table 6.** Reconstructed Average Daily Diet with Daily Serving of Foods, Including Their Protein and fat Contributions, in Grams (Arnold et al. 2006; Mol and Turan 2008; Moss 2016; I. Smith 2011; M. Smith 2018).

Group	Results			Retrieved data					
	Daily serving (g)	Percent of whole	Live amount per family of 4 per week^a^	gProtein/100g	Protein daily serving (g)	gFat/100g	Fat daily serving (g)	Live weight in kg^b^	Edible percentage out of 1.00
*Land mammals*									
Ungulates	15.00	1.14	0.01	22.72	3.41	1.53	0.23	93.30	0.55
Sm terrestrial mammals	10.00	0.76	0.03	146	14.60	4.80	0.48	20.50	0.50
*Plants*									
Berries	10.00	0.76	0.028 kg	0.9	0.09	0.51	0.05	-	1.00
Roots	10.00	0.76	0.028 kg	5.3	0.53	0.29	0.03	-	1.00
Plant foods	10.00	0.76	0.028 kg	1.8	0.18	0.51	0.05	-	1.00
Fungi	5.00	0.38	0.014 kg	1.5	0.08	0.53	0.03	-	1.00
*Birds*	150.00	11.43	5.22	11.49	17.24	39.34	59.01	1.20	0.67
*Marine mammals*									
Sea lions	9.00	0.69	0.00001	24.25	2.18	9.25	0.93	487.50	0.54
Seals	1.45	0.11	0.001	20.5	0.3	3.20	1.44	93.50	0.38
*Fish*									
Marine white fishes	50.00	3.81	0.30	19.09	9.55	5.86	2.93	6.76	0.70
Flatfish	48.00	3.66	1.47	15.485	7.43	1.63	0.78	1.33	0.69
Herring spawn	75.00	5.71	2.1 kg	11.3	8.48	0.80	0.60	0.01	1.00
Herring	175.00	13.33	50	16.4	28.70	13.88	24.29	0.14	0.70
Spiny dogfish	15.00	1.14	0.24	20.98	3.15	4.51	0.68	4.87	0.36
Sturgeon	30.00	2.29	0.03	16.1	4.83	4.04	0.59	38.94	0.62
Pink salmon	32.40	2.47	0.47	20.5	6.64	4.40	1.90	2.63	0.73
Chum salmon	176.85	13.33	1.62	20.1	35.55	3.77	8.89	4.14	0.74
Sockeye salmon	1.96	0.15	0.02	21.3	0.42	5.61	1.46	4.14	0.75
Coho salmon	5.90	0.45	0.05	21.6	1.27	5.93	0.47	4.26	0.75
Chinook salmon	1.96	0.15	0.02	19.9	0.39	10.43	2.72	4.26	0.72
Salmonid	4.91	0.37	0.05	20.77	1.02	6.03	0.39	3.65	0.74
Salmon roe	40.00	3.05	1.12 kg	26.80	10.72	14.00	5.60	0.01	1.00
Anchovy	75.00	5.71	105	20.4	15.30	4.84	3.63	0.02	1.00
Eulachon	50.00	3.81	28	20.5	10.25	24.80	6.20	0.05	1.00
Surf smelt	75.00	5.71	42	17.6	13.20	2.42	1.82	0.05	1.00
Surfperch	5.00	0.38	0.62	19.4	0.97	0.92	0.05	0.50	0.45
Rockfish	5.00	0.38	0.14	18.4	0.92	1.34	0.07	1.77	0.57
Sculpin	5.00	0.38	0.42	18.4	0.92	1.34	0.07	0.85	0.39
Stickleback	5.00	0.38	15.56	17.66	0.88	1.06	0.05	0.01	0.90
*Tidal and other foods*									
Crabs	50.00	3.81	5.38	17.4	8.70	0.97	0.15	1.04	0.25
Bivalves	150.00	11.43	39.22	14.7	22.05	0.96	1.68	0.63	0.17
Echinoderms	5.00	0.38	0.14 kg	13	0.65	0.40	0.02	0.51	0.23
Octopus/squid	5.00	0.38	6.57	15.6	0.78	1.38	0.07	5.00	0.71
Shrimp	5.00	0.38	10.37	13.6	0.68	1.01	0.05	0.03	0.45
*Totals*	**1312**.**42**	** 100**			**232**.**04**		**126**.**04**		

The daily servings are averaged across seasons. Edible percentage includes the edible portion after removing any inedible portion (Ashley 2002; Crapo, Paust and Babbitt 1993; Moss 2016). Weekly amount is in amount of food (live weight or number/amount of live animals) consumed per family of four per week, unless measured in kg, in which case it is amount in kg per family of four per week.

^a^ Unless labeled in kg, amount represents number of live animals (i.e., 0.04 indicates 4 percent of a live animal).

^b^ Live weight in kg is averaged across adult male and adult female averages of each food group.

The precontact səl̓ilwətaɬ diet reconstruction incorporates all data sources and deliberately included a focus on the four main pillars of the səl̓ilwətaɬ diet (salmon, forage fish, shellfish, and marine birds). Incorporation of səl̓ilwətaɬ traditional ecological and cultural knowledge and səl̓ilwətaɬ community history provided much-needed refinement to ensure the diet was reasonable and reflective of the knowledge passed down through generations.

## Discussion

This research provides a novel approach to traditional dietary reconstruction and a greater resolution in our understanding of traditional səl̓ilwətaɬ diets by using a collaborative ethnohistorical framework and combining multiple interdisciplinary data sources. Another benefit of this approach is the avoidance of the destructive analysis of səl̓ilwətaɬ Ancestral Remains using stable isotope analysis. We do not include imported foods, as our focus is on reconstructing the diet that the ecosystem of səl̓ilwət offered, which would have constituted the majority of precontact səl̓ilwətaɬ diets. However, as discussed above, the exchange of food including the import and export of foods beyond səl̓ilwət is an important component of Coast Salish culture and kinship (H. G. Barnett 1955; Suttles 1960), and imported foods could be considered in future research.

Including səl̓ilwətaɬ TEK was an essential part of the approach we took in this study, and provided essential data and context that allowed us to create a realistic best-estimate of a precontact səl̓ilwətaɬ diet. Incorporating the three limiting factors (local ecology, nutritional needs, and dietary focus) offers boundaries in the dietary reconstruction that both accommodates for disciplinary biases and allows for best possible estimate. This interdisciplinary approach provides a more holistic and realistic understanding than faunal archeology alone could. This research thus showcases a new methodology for dietary reconstructions of ancestral communities that can be adapted and applied in other contexts.

Our method provides a robust working hypothesis or best estimate of a precontact səl̓ilwətaɬ diet. It does not, however, mean that this was the diet for everyone, all the time. There would have certainly been significant seasonal variations in the range of foods eaten by everyone, some people may have had permanent or short proscriptions against eating certain foods on account of gender or life-crisis ceremonies. Additionally, there could have been differences in the diets of people of different social status and roles/professions, and long-term ecological changes that could have resulted in dietary changes. Finally, we do not consider imported foods, such as sockeye salmon from the Fraser River, which would have been processed shortly after fishing and the bones would not be represented in the archeological sites on the shores of səl̓ilwət. The diet we present above is an average precontact səl̓ilwətaɬ diet that does not include seasonal and cultural differences.

By reconstructing what the traditional səl̓ilwətaɬ diet could have been, we have created a baseline from which səl̓ilwətaɬ can plan future nutritional reliance on the səl̓ilwət ecosystem. This research aids in the understanding of səl̓ilwətaɬ communities who lived in and relied on the səl̓ilwət ecosystem. The reconstructed diet showcases səl̓ilwətaɬ marine and tidal resource reliance, and the variety contained within the annual diet. The diet had to fill the caloric needs of the ancestral səl̓ilwətaɬ population, following the traditional knowledge of what foods would have been eaten. The high fat content allows for the safe consumption of the high protein content, and together they provide an energy-dense diet that would have been essential for a highly active population. While berries, green foods, and root vegetables are part of the diet, they do not provide many calories compared to more calorically dense foods, such as fish, fish oils, and meat. These plant foods are going to be somewhat hidden in a dietary analysis that looks at primarily protein and fats.

## Conclusions

This is, to the best of our knowledge, the first attempt to reconstruct a traditional diet using this interdisciplinary approach in the Pacific Northwest. As such, it is the best estimate of the precontact diet based on the available data. Marine and tidal resources form the heart of traditional səl̓ilwətaɬ diet. The precontact səl̓ilwətaɬ diet was varied and relied on land- and waterscape stewardship of resources and ecosystems to be sustainable over generations. This approach to reconstructing precontact Indigenous diets reminds us to consider archeologically invisible parts of the diet, such as octopus, sea cucumber, and many plant foods. Archeological efforts that do not consider these archeologically invisible parts of the ecosystem do not show the whole picture. Any one discipline is going to have gaps in its data, and by combining data from multiple disciplines and sources, we seek to avoid data gaps and build a robust estimate of the precontact diet. This diet offers a data-driven, interdisciplinary perspective on səl̓ilwətaɬ subsistence and relationships with their ancestral territory.

We know from previous work that səl̓ilwətaɬ communities would have been eating across the ecosystem (Pierson 2011; Morin 2015; Tsleil-Waututh Nation, 2016; Morin et al. 2018; Guttmann 2022; Morin and Evans 2022), which would have allowed for seasonal shifts and more sustainable fishing, harvesting, and hunting practices. Reconstruction of precontact diets aids in the understanding of the past and of the relationships between human communities and their home environments. By reconstructing precontact diet of səl̓ilwətaɬ communities, we now have a better understanding of səl̓ilwətaɬ Ancestors and their subsistence. This diet offers a window into the subsistence practices of the ancestral səl̓ilwətaɬ communities and showcases not only the fish, marine, and tidal specialization that is so central to səl̓ilwətaɬ culture, subsistence, and stewardship, but also fishing, hunting, and harvesting across the ecosystem, increasing sustainability and variety.

## Supplemental Material

sj-docx-1-ebi-10.1177_02780771241261235 - Supplemental material for A Fish-Focused Menu: An Interdisciplinary Reconstruction of Ancestral Tsleil-Waututh Diets Supplemental material, sj-docx-1-ebi-10.1177_02780771241261235 for A Fish-Focused Menu: An Interdisciplinary Reconstruction of Ancestral Tsleil-Waututh Diets by Meaghan Efford, Santiago de la Puente, Micheal George, Michelle George, Alessandria Testani, Spencer Taft, Jesse Morin, Jay Hilsden, Jennifer Zhu, Pengpeng Chen, Lindsey Paskulin, Ginevra Toniello, Villy Christensen and Camilla Speller in Journal of Ethnobiology
