# Supplementary material for: A Fish-Focused Menu: An Interdisciplinary Reconstruction of Ancestral Tsleil-Waututh Diets
Source: J Ethnobiol. 2024 Aug 2;44(3):247–63. doi: 10.1177/02780771241261235 (PMC13021004; doi:10.1177/02780771241261235)
Supplement: sj-docx-1-ebi-10.1177_02780771241261235 - Supplemental material for A Fish-Focused Menu: An Interdisciplinary Reconstruction of Ancestral Tsleil-Waututh Diets [file sj-docx-1-ebi-10.1177_02780771241261235.docx]

**Supplementary information for**

# **A fish-focused menu: an interdisciplinary reconstruction of Ancestral Tsleil-Waututh Diets**

**Biomolecular Methods**

*Zooarchaeology by Mass Spectrometry (ZooMS)*

Taxonomic identifications of 52 samples were undertaken using collagen peptide mass fingerprinting (or ZooMS) following Buckley et al. [(2009)](https://paperpile.com/c/pxEfRW/dFwn/?noauthor=1) and modified as in Rodrigues et al. [(2018)](https://paperpile.com/c/pxEfRW/8vmt/?noauthor=1) (Supplementary Table 1). Bones were subsampled and collagen was extracted within the Ancient DNA and Proteins (ADαPT) Laboratories at the University of British Columbia. The bone samples were demineralized in a weak acid solution (0.6M HCl); the sample was then centrifuged, and the supernatant was discarded. The samples were rinsed with 250 µL of 0.1M NaOH, and then rinsed three times with 200 μl of 50 mMol ammonium bicarbonate, pH 8.0 (AmBic solution), and gelatinized by heating at 65º C in AmBic solution for 1 hour. The collagen was enzymatically cleaved with trypsin at 37˚C, and purified using 100 µl Pierce ™ C18 pipette tips. Equal amounts of the collagen extract and α-cyano-hydroxycinnamic acid matrix solution (1% in conditioning solution) were mixed and spotted in triplicate onto a 384 spot MALDI target plate, with calibration standards. Samples were run on a Bruker ultraflex III MALDI TOF/TOF mass spectrometer with a Nd:YAG smart beam laser. We used the mMass software (Strohalm et al. 2008) to average spectra from replicates, and compare them to published *m/z* markers Buckley et al. [(2009)](https://paperpile.com/c/pxEfRW/dFwn/?noauthor=1), Buckley and Collins [(2011)](https://paperpile.com/c/pxEfRW/kC3m/?noauthor=1), Buckley et al. [(2014)](https://paperpile.com/c/pxEfRW/o8B7/?noauthor=1), Buckley et al. [(2017)](https://paperpile.com/c/pxEfRW/Lczz/?noauthor=1), Buckley et al. [(Buckley et al. 2022)](https://paperpile.com/c/pxEfRW/HdjX), Codlin et al. [(2022)](https://paperpile.com/c/pxEfRW/hjd8/?noauthor=1), Dierickx et al. [(2022)](https://paperpile.com/c/pxEfRW/Ebvi/?noauthor=1), Kirby et al. [(2013)](https://paperpile.com/c/pxEfRW/Gsc4/?noauthor=1), McGrath et al. [(2019)](https://paperpile.com/c/pxEfRW/ssy8/?noauthor=1), Korzow-Richter et al. [(2020)](https://paperpile.com/c/pxEfRW/6GiQ/?noauthor=1), Welker et al. [(2016)](https://paperpile.com/c/pxEfRW/bqOh/?noauthor=1). Taxonomic identifications were assigned at the most conservative level of identification (species, genus, or family level) based on the presence of unambiguous *m/z* markers. ‘Probable’ identifications were assigned if spectra presented observable diagnostic species markers below the signal to noise ratio of 6.0. [Note: MALDI raw spectra for all samples will be available in the Dryad Digital Repository following manuscript acceptance at DOI: 10.5061/dryad.ns1rn8q10].

**Supplementary Table 1: Sample information, provenience and ZooMs identification of faunal samples from təmtəmíxʷtən (DhRr-6)**

| **ADαPT Lot Number** | **Sample Name** | **Provenience** | **Weight (mg)** | **ZooMS ID** |
| --- | --- | --- | --- | --- |
| A832 | L3 S1 | 116-118 N 4-6 W Level 3 | 23 | No ID |
| A833 | L5 S2 | 116-118 N 4-6 W Level 5 | 26 | Beaver (*Castor* sp.) |
| A834 | L5 S3 | 116-118 N 4-6 W Level 5 | 26 | Bovid/Cervid |
| A835 | L5 S4 | 116-118 N 4-6 W Level 5 | 19 | Elk/Moose (*Cervus/Alces*) |
| A836 | L6 S5 | 116-118 N 4-6 W Level 6 | 27 | No ID |
| A837 | L6 S6 | 116-118 N 4-6 W Level 6 | 15 | Deer (*Odocoileus sp.*)/Sheep |
| A838 | L9 S7 | 116-118 N 4-6 W Level 9 | 27 | Beaver (*Castor* sp.) |
| A839 | L9 S8 | 116-118 N 4-6 W Level 9 | 25 | Deer (*Odocoileus sp.*) |
| A840 | L9 S9 | 116-118 N 4-6 W Level 9 | 25 | Deer (*Odocoileus sp.*) - probable |
| A841 | L9 S10 | 116-118 N 4-6 W Level 9 | 22 | Bird (Aves) |
| A842 | L9 S11 | 116-118 N 4-6 W Level 9 | 30 | Deer (*Odocoileus sp.*) - probable |
| A843 | L12 S12 | 116-118 N 4-6 W Level 12 | 21 | Bovid/Cervid |
| A844 | L12 S13 | 116-118 N 4-6 W Level 12 | 18 | Deer (*Odocoileus sp.*) |
| A845 | L14 S14 | 116-118 N 4-6 W Level 14 | 20 | Bovid/Cervid |
| A846 | L14 S15 | 116-118 N 4-6 W Level 14 | 30 | Seal (Phocinae) |
| A847 | L14 S16 | 116-118 N 4-6 W Level 14 | 21 | Seal (Phocinae) |
| A848 | L15 S17 | 116-118 N 4-6 W Level 15 | 24 | Bovid/Cervid |
| A849 | L15 S18 | 116-118 N 4-6 W Level 15 | 27 | Bear (Ursidae) |
| A850 | L15 S19 | 116-118 N 4-6 W Level 15 | 20 | Seal (Phocinae) |
| A851 | L15 S20 | 116-118 N 4-6 W Level 15 | 17 | Musteloid (Musteloidea) |
| A852 | L15 S21 | 116-118 N 4-6 W Level 15 | 22 | Seal (Phocinae) |
| A853 | L16 S22 | 116-118 N 4-6 W Level 16 | 19 | Carnivora |
| A854 | L17 S23 | 116-118 N 4-6 W Level 17 | 28 | Carnivora |
| A855 | L17 S24 | 116-118 N 4-6 W Level 17 | 24 | Bird (*Anatidae*) |
| A856 | L7 P1 | Pit 1A Level 7 | 27 | Deer (*Odocoileus sp.*) - probable |
| A857 | L7 P2 | Pit 1A Level 7 | 26 | Deer (*Odocoileus sp.*) - probable |
| A858 | L7 P3 | Pit 1A Level 7 | 23 | Deer (*Odocoileus sp.*) - probable |
| A859 | L7 P4 | Pit 1A Level 7 | 23 | Dolphin (*Dolpheninae*) |
| A860 | L8 P1 | Pit 1A Level 8 | 18 | Deer (*Odocoileus sp.*) |
| A861 | L8 P2 | Pit 1A Level 8 | 19 | Seal (Phocinae) |
| A862 | L8 P3 | Pit 1A Level 8 | 23 | Elk/Moose (*Cervus/Alces*) |
| A863 | L8 P4 | Pit 1A Level 8 | 24 | Deer (*Odocoileus sp.*) - probable |
| A864 | L8 P5 | Pit 1A Level 8 | 19 | Deer (*Odocoileus sp.*) - probable |
| A865 | L8 P6 | Pit 1A Level 8 | 18 | Deer (*Odocoileus sp.*) - probable |
| A866 | L8 P7 | Pit 1A Level 8 | 16 | Deer (*Odocoileus sp.*) - probable |
| A867 | L12 P1 | Pit 1A Level 12 | 20 | Deer (*Odocoileus sp.*) - probable |
| A868 | L12 P2 | Pit 1A Level 12 | 27 | Canid (*Canis* sp.) |
| A869 | L13 P1 | Pit 1A Level 13 | 18 | Deer (*Odocoileus sp.*) - probable |
| A870 | L13 P2 | Pit 1A Level 13 | 21 | Beaver (*Castor* sp.) |
| A871 | L16 P1 | Pit 1A Level 16 | 17 | Beaver (*Castor* sp.) |
| A872 | L16 P2 | Pit 1A Level 16 | 21 | Musteloid (Musteloidea) |
| A873 | L16 P3 | Pit 1A Level 16 | 26 | Deer (*Odocoileus sp.*) - probable |
| A874 | L16 P4 | Pit 1A Level 16 | 16 | Seal (Phocinae) |
| A875 | L16 P5 | Pit 1A Level 16 | 30 | No ID |
| A876 | L16 P6 | Pit 1A Level 16 | 24 | Bird (Aves) |
| A877 | L8 X1 | 116-118 N 0-2 W Level 8 | 16 | Starry flounder (*Platichthys stellatus*) - probable |
| A878 | L8 X2 | 116-118 N 0-2 W Level 8 | 16 | Bird (Anatidae) |
| A879 | L8 X3 | 116-118 N 0-2 W Level 8 | 19 | Rockfish (*Sebastes sp.*) - probable |
| A880 | L8 X4 | 116-118 N 0-2 W Level 8 | 20 | Elk/Moose (*Cervus/Alces*) |
| A881 | L8 X5 | 116-118 N 0-2 W Level 8 | 20 | Humpback whale (*Megaptera novaeangliae*) |
| A882 | L7 X1 | 116-118 N 0-2 W Level 7 | 24 | Rockfish (*Sebastes sp.*) - probable |
| A883 | L7 X2 | 116-118 N 0-2 W Level 7 | 27 | Starry flounder (*Platichthys stellatus*) - probable |

*Ancient DNA Extraction*

Ancient DNA analysis was conducted on a subset of 17 samples to confirm ZooMS identifications, or provide more precise identifications. Sample preparation and DNA extraction was conducted in the ADαPT dedicated biomolecular cleanroom using a silica spin column method [(Yang et al. 1998)](https://paperpile.com/c/pxEfRW/srOD) modified as in Rodrigues et al. [(Rodrigues et al. 2018)](https://paperpile.com/c/pxEfRW/8vmt) and following vigorous contamination control protocols [(Yang and Watt 2005)](https://paperpile.com/c/pxEfRW/hjma). Approximately 30-120 mg of each bone sample was subsampled and chemically decontaminated through submersion in 6% sodium hypochlorite, followed by UV irradiation for 20 min each on two sides. The subsamples were crushed into powder and incubated overnight in a lysis buffer (0.5 M EDTA pH 8.0; 0.5 mg/mL proteinase K) in a rotating hybridization oven at 50°C. Samples were then centrifuged and 1.8 mL of supernatant from each sample was concentrated to 100 μL using Amicon Ultra-4 Centrifugal Filter Devices (10 KD, 4mL, Millipore). Concentrated extracts were purified using QIAquick minelute columns (QIAGEN, Hilden, Germany) and 80 μL of DNA solution was eluted from each QIAquick column for PCR amplification.

## *Ancient DNA Species Identification*

Species identification was based on phylogenetic analyses of mitochondrial DNA fragments, including the 12S rRNA gene and control region (D-loop), following PCR conditions described in Speller et al. [(Speller et al. 2014)](https://paperpile.com/c/pxEfRW/1lKY). PCR was undertaken using 4 different primer sets:

1. primers Cervid-12S-F70 and Cervid-12S-R193 targeted a second 123 bp fragment of mammal 12SrRNA [(Speller et al. 2014)](https://paperpile.com/c/pxEfRW/1lKY).
2. primers Cervid-12S-F70 and Cervid-12S-R250 targeted a longer 180 bp fragment of mammal 12SrRNA [(Speller et al. 2014)](https://paperpile.com/c/pxEfRW/1lKY).
3. primers Mam-12S-F261 [5’- CCACCGCGGTCATACGATT-3’] and Mam-12S-R460 [5’-GCATAGTGGGGTATCTAATCCCAG-3’] targeted a second 199 bp fragment of mammal 12SrRNA.
4. primers F29ddlp [5’-AACACCCAAAGCTGAAGTTCTATT-3’] and R316ddlp [5’-ACTTGCTTATAAGYATGGGGTATATAAT-3’] targeted a 287 bp fragment of control region (D-loop) in *Odocoileus* sp. [(Clark 2023)](https://paperpile.com/c/pxEfRW/p1Hc)

Five uL of PCR product from each PCR amplification were separated on a 2% agarose gel, and visualized using SYBR Green. The results of the PCR amplifications and sequencing can be found on Supplementary Table 2. Successfully amplified samples were sent to Eurofins Genomics Ltd. for sequencing using the forward and/or reverse primers.The obtained sequences were compared to Genbank sequences through the BLAST application to determine their closest match, and to ensure that they did not match with any other unexpected species or sequences. Sample sequences were visually edited and base pair ambiguities were examined using ChromasPro software (www.technelysium.com.au). The 12S rRNA fragments were truncated to remove the primer sequences, and combined to produce overlapping consensus fragments for phylogenetic analysis. Multiple alignments of ancient sequences and published cervid reference sequences were conducted using ClustalW [(Thompson et al. 1994)](https://paperpile.com/c/pxEfRW/KeFv), through BioEdit [(Hall 2001)](https://paperpile.com/c/pxEfRW/qh0O). Phylogenetic analyses were performed through MEGA 11 [(Tamura et al. 2021)](https://paperpile.com/c/pxEfRW/XYVz).

**Supplementary Table 2 - Sample, provenience, and identification of archaeological faunal remains from təmtəmíxʷtən (DhRr-6) using aDNA**

| **ADαPT Lot Number** | **Sample Name** | **Provenience** | **Weight analyzed (mg)** | **Taxonomic ID (haplotype)** |
| --- | --- | --- | --- | --- |
| A834 | L5 S3 | 116-118 N 4-6 W Level 5 | 116 | Failed |
| A835 | L5 S4 | 116-118 N 4-6 W Level 5 | 132 | *Cervus canadensis* |
| A837 | L6 S6 | 116-118 N 4-6 W Level 6 | 48 | *Odocoileus hemionus* |
| A839 | L9 S8 | 116-118 N 4-6 W Level 9 | 117 | *Odocoileus hemionus* |
| A840 | L9 S9 | 116-118 N 4-6 W Level 9 | 75 | *Odocoileus hemionus* |
| A842 | L9 S11 | 116-118 N 4-6 W Level 9 | 103 | Failed |
| A843 | L12 S12 | 116-118 N 4-6 W Level 12 | 129 | *Cervus canadensis* |
| A845 | L14 S14 | 116-118 N 4-6 W Level 14 | 110 | *Oreamnos americanus* |
| A848 | L15 S17 | 116-118 N 4-6 W Level 15 | 123 | *Cervus canadensis* |
| A851 | L15 S20 | 116-118 N 4-6 W Level 15 | 81 | Failed |
| A853 | L16 S22 | 116-118 N 4-6 W Level 16 | 93 | Failed |
| A854 | L17 S23 | 116-118 N 4-6 W Level 17 | 119 | Failed |
| A862 | L8 P3 | Pit 1A Level 8 | 113 | *Cervus canadensis* |
| A865 | L8 P6 | Pit 1A Level 8 | 38 | *Odocoileus hemionus* |
| A866 | L8 P7 | Pit 1A Level 8 | 81 | Failed |
| A872 | L16 P2 | Pit 1A Level 16 | 50 | *Mephitis mephitis* |
| A880 | L8 X4 | 116-118 N 0-2 W Level 8 | 114 | *Cervus canadensis* |

***ZooMS Results***

Of the 52 bone samples provided, 49 were successfully identified to various taxonomic levels. The most commonly identified taxa were cervids. Fifteen samples were identified as deer (*Odocoileus sp*) (Supplementary Figure 1). Three samples were identified as either North American elk or moose since ZooMS is not capable of distinguishing elk (*Cervus elphus*), moose (*Alces alces*) or the European fallow deer (*Dama dama*) [(von Holstein et al. 2014)](https://paperpile.com/c/pxEfRW/yy2J) (Supplementary Figure 1). Due to a lack of diagnostic markers, five samples could only be identified to broader taxonomic categories, like *Bovid/Cervid*. These five samples are likely cervids, however they were missing a distinctive C _ ɑ2 502 marker which can differentiate between the two families.

Six samples were identified as family Phocidae (earless seals), specifically the Phocini tribe. In this archaeological context, these samples likely represent common or harbour seal (*Phoca vitulina*). Four samples were identified as birds which currently cannot be differentiated any further than the family level using ZooMS due to their evolutionarily conserved collagen sequence and the lack of ZooMS reference databases specific to birds [(Codlin et al. 2022)](https://paperpile.com/c/pxEfRW/hjd8). Two of these samples (A855 and A878) could be identified to the family Anatidae which includes taxa like ducks, geese, and swans (Codlin et al., 2022), however the remaining two samples yielded collagen fingerprints that could not be matched to a particular family or order. Four samples were identified as non-salmon fish (spectra for sample A877 matches that of A883; spectra for sample A879 matches that of A882). A877 and A883 were identified as European flounder (*Plaitchthys flesus*) using reference databases from Dierickx et al. [(2022)](https://paperpile.com/c/pxEfRW/Ebvi/?noauthor=1) and Buckley et al. [(2022)](https://paperpile.com/c/pxEfRW/HdjX/?noauthor=1); however this fish is only found in Atlantic waters, and so we suggest a possible identification as Starry flounder (*Platicthys stellatus*) given their close taxonomic relationship and the fact that starry flounder is the only member of the genus *Platichthys* to inhabit Pacific waters [(see Vinnikov et al. 2018)](https://paperpile.com/c/pxEfRW/Dmt4). A879 and A882 were identified as Norway redfish (*Sebastes viviparus*) using the Buckley et al. [(2022)](https://paperpile.com/c/pxEfRW/HdjX/?noauthor=1) reference database. Similar to the European founder, Norway redfish is an Atlantic species; thus, we suggest these samples likely represent a Pacific rockfish species (*Sebastes sp.*) not yet characterized by ZooMS. Four samples were identified as beaver (*Castor canadensis*; Supplementary figure 2), two as musteloids (superfamily Musteloidea), and two as carnivora. The musteloid specimens (e.g., weasels, otters, badgers, and skunks) could only be identified to the superfamily level since they lack a direct match in the database, which is currently confined to European species. The two spectra for the samples identified as “carnivora” had few diagnostic peaks, making further taxonomic precision difficult. One sample was identified as a canid (*Canis* sp), representing domestic dog (*Canis familiaris*), grey wolf (*Canis lupus*) or coyote (*Canis latrans*). One sample was identified as the subfamily Ursinae, which may represent black bear (*Ursus americanus*) or brown bear (*Ursus arctos*), which cannot be distinguished through ZooMS [(Buckley et al. 2009)](https://paperpile.com/c/pxEfRW/dFwn). One sample was identified as a dolphin, specifically subfamily Delphininae, and one other as a humpback whale (*Megaptera novaeangliae*).

**Supplementary Figure 1 Example spectra for elk/ moose (*Cervus/Alces*) (top) and deer (*Odocoileus* sp.) bottom, with key markers for identification in red font.**

# **
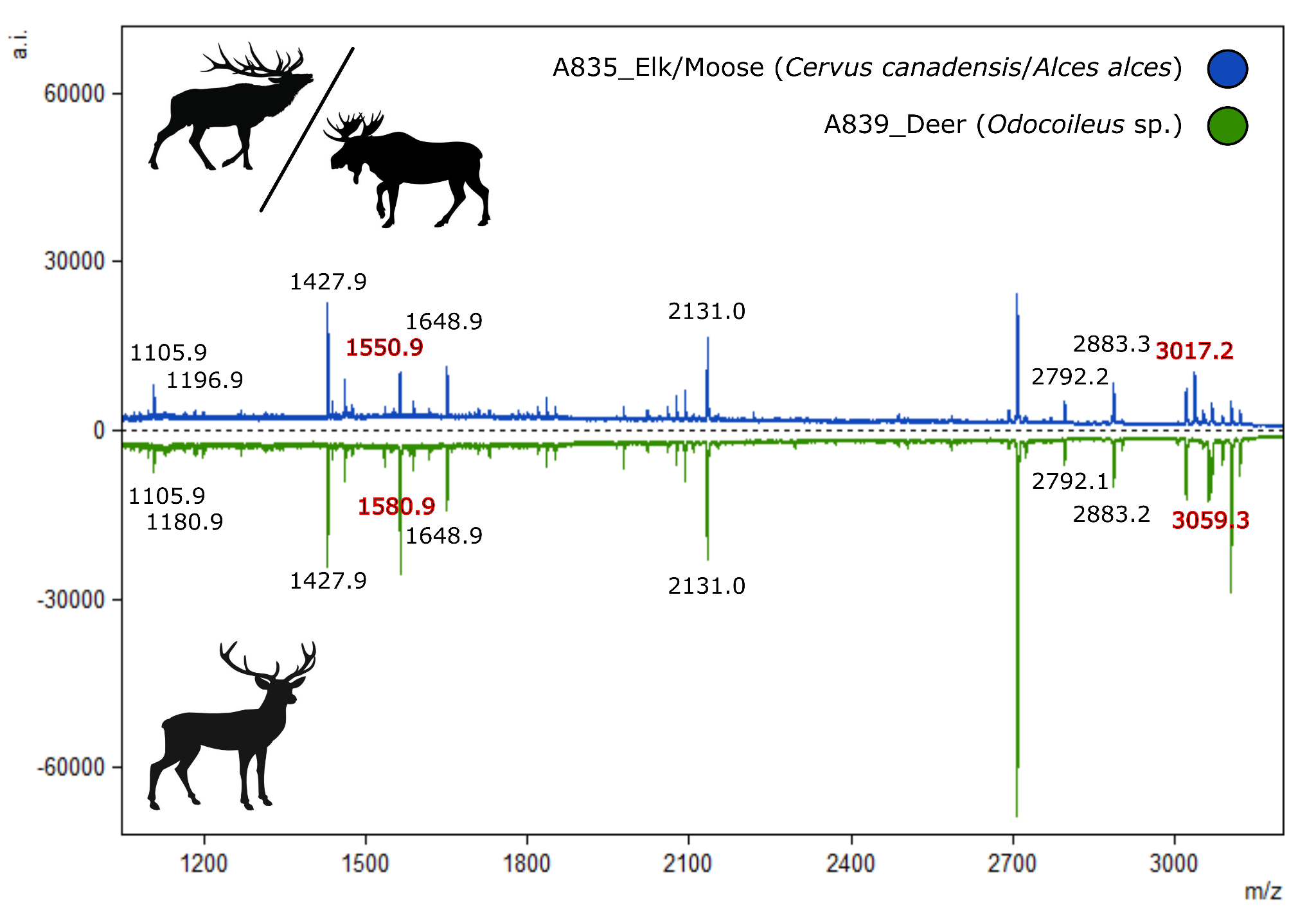
**

**Supplementary Figure 2 Example spectra for beaver (*Castor canadensis*), with key markers for identification.**

**
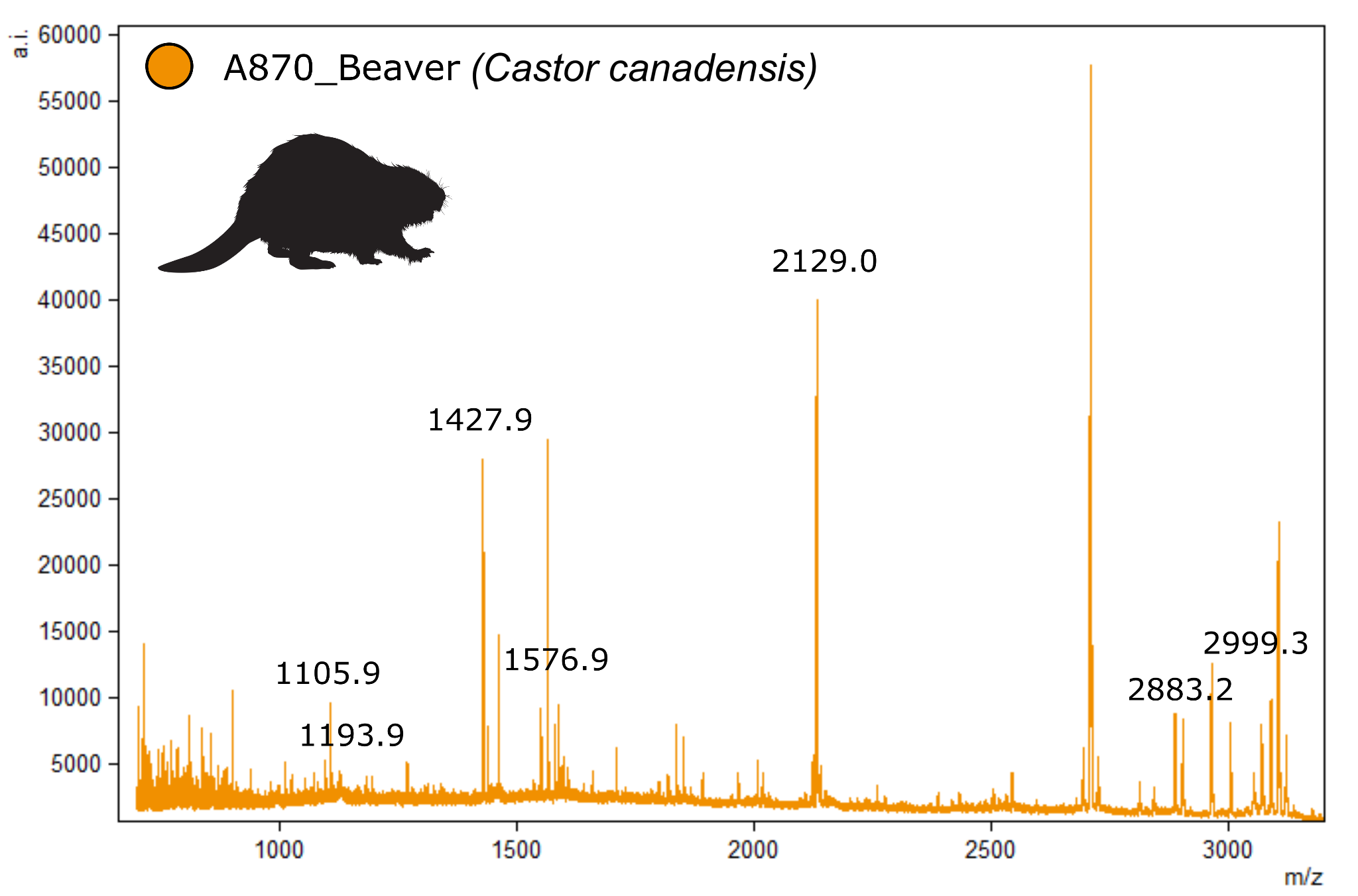
**

***Ancient DNA Results***

Of the 17 tested samples, 11 could be identified to species through ancient mitochondrial DNA analysis. One of the Musteloidea samples (A872) could be confidently assigned to striped skunk (*Mephitis mephitis*) (Supplementary Figure 3). The other Musteloid sample and the two carnivore samples identified through ZooMS did not produce successful PCR amplifications. This is most likely due to poor DNA preservation in these samples, although primer incompatibility may also be an issue.

The 12S rRNA fragment of mtDNA could confidently identify five samples as North American elk (*Cervus canadensis,* also known as *Cervus elaphus*) and one sample (A845) as mountain goat (*Oreamnos americanus*) (Supplementary Figure 4). This fragment of mtDNA, however, could not confidently identify four of the samples, in part due to a lack of sequence diversity in the 12S rRNA gene among *Odocoileus* sp. and due to DNA damage in the recovered sequence for A839. Subsequently, these four samples were amplified with a primer set designed to target the hypervariable control region (D-loop) of *Odocoileus* sp. [(Clark 2023)](https://paperpile.com/c/pxEfRW/p1Hc). The control region data matched most closely with published *Odocoileus hemionus* (Black-tailed deer) sequences [(Latch et al. 2009)](https://paperpile.com/c/pxEfRW/RPOA).

**
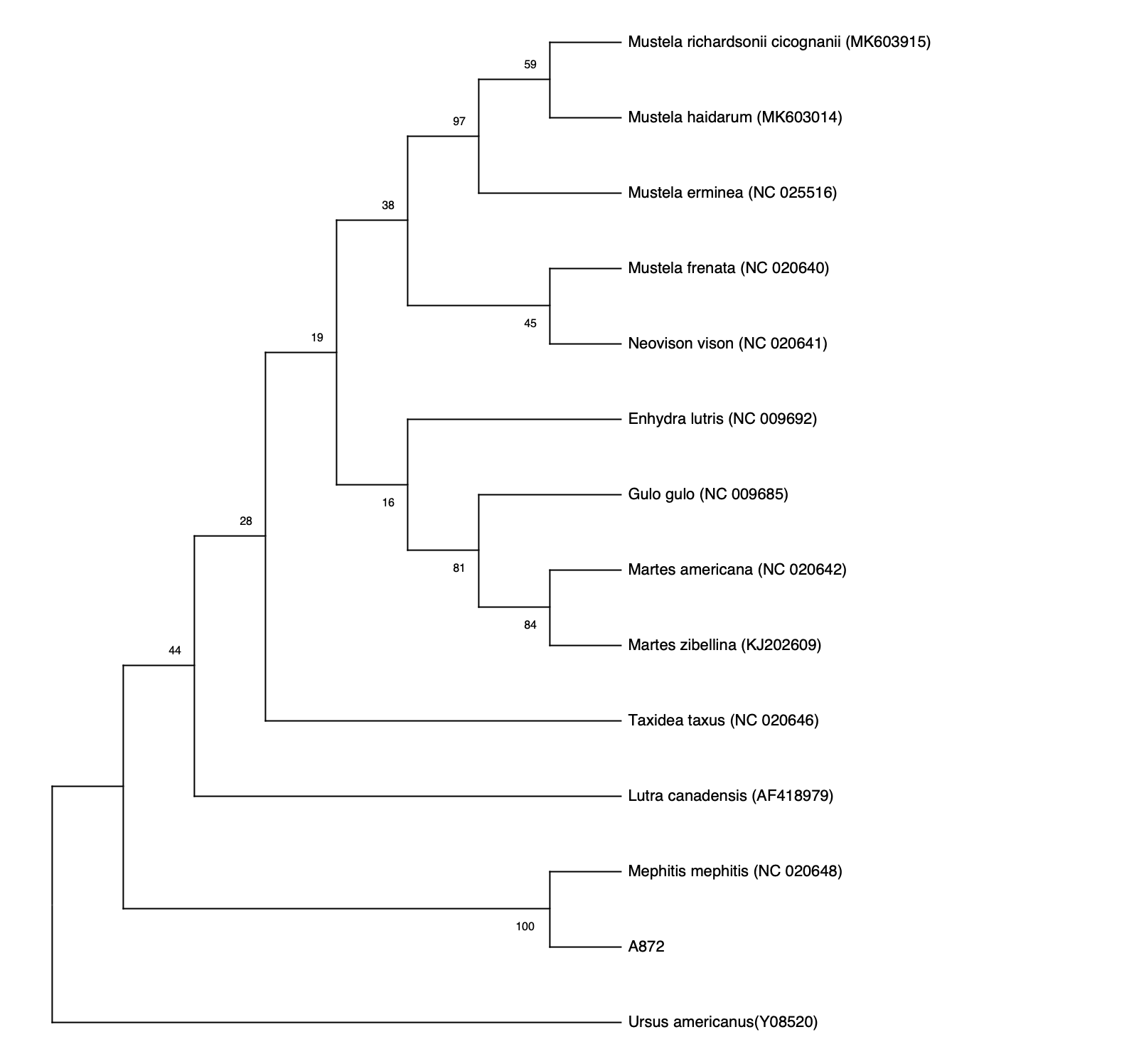
**

**Supplementary Figure 3 Phylogenetic analysis of modern Musteloidea species and A872 using 118bp of 12S rRNA with *Ursus americanus* as the outgroup. The bootstrap consensus tree is inferred from 500 replicates using using the Neighbor-Joining method with distances computed using the Kimura 2-parameter method.**


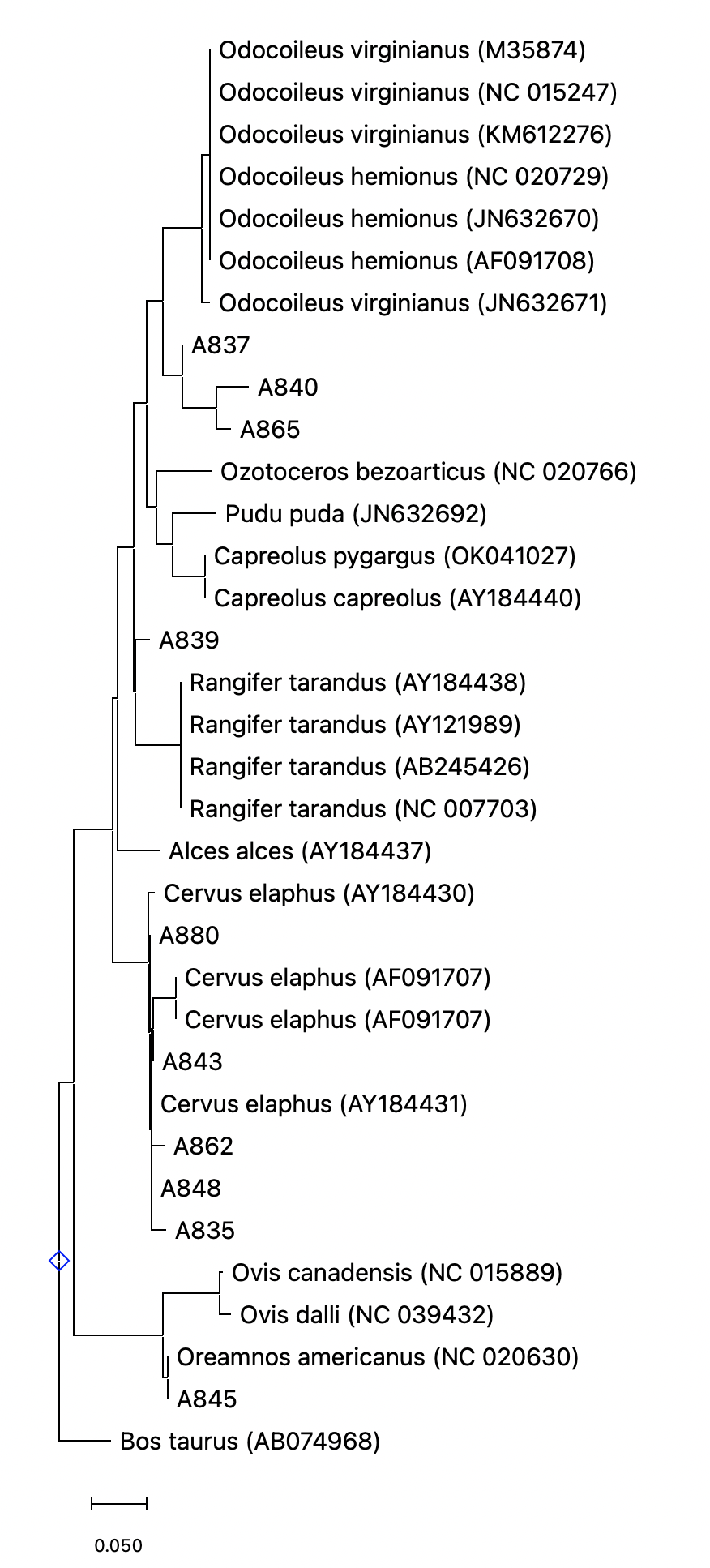


**Supplementary Figure 4 Phylogenetic analysis of modern and ancient bovids and cervids using 142bp of 12S rRNA. The bootstrap consensus tree is inferred using the Neighbor-Joining method with distances computed using the Kimura 2-parameter method.**

**References**

[Buckley, M., and M. J. Collins. 2011. Collagen Survival and Its Use for Species Identification in Holocene-Lower Pleistocene Bone Fragments from British Archaeological and Paleontological Sites. *Antiqua* 1:e1–e7.](http://paperpile.com/b/pxEfRW/kC3m)

[Buckley, M., M. Collins, J. Thomas-Oates, and J. C. Wilson. 2009. Species Identification by Analysis of Bone Collagen Using Matrix-Assisted Laser Desorption/ionisation Time-of-Flight Mass Spectrometry. *Rapid communications in mass spectrometry: RCM* 23:3843–3854.](http://paperpile.com/b/pxEfRW/dFwn)

[Buckley, M., S. Fraser, J. Herman, N. D. Melton, J. Mulville, and A. H. Pálsdóttir. 2014. Species Identification of Archaeological Marine Mammals Using Collagen Fingerprinting. *Journal of archaeological science* 41:631–641.](http://paperpile.com/b/pxEfRW/o8B7)

[Buckley, M., V. L. Harvey, and A. T. Chamberlain. 2017. Species Identification and Decay Assessment of Late Pleistocene Fragmentary Vertebrate Remains from Pin Hole Cave (Creswell Crags, UK) Using Collagen Fingerprinting. *Boreas* 46:402–411.](http://paperpile.com/b/pxEfRW/Lczz)

[Buckley, M., V. L. Harvey, D. Petiffer, H. Russ, W. Wouters, and W. Van Neer. 2022. Medieval Fish Remains on the Newport Ship Identified by ZooMS Collagen Peptide Mass Fingerprinting. *Archaeological and anthropological sciences* 14:41.](http://paperpile.com/b/pxEfRW/HdjX)

[Clark, L. 2023. *Oh Deer: Ancient DNA Analysis of Archaeological Deer Remains from Housepit 54, Bridge River Archaeological Site (EeRI-4), British Columbia, Canada*. Masters Thesis, Simon Fraser University.](http://paperpile.com/b/pxEfRW/p1Hc)

[Codlin, M. C., K. Douka, and K. K. Richter. 2022. An Application of Zooms to Identify Archaeological Avian Fauna from Teotihuacan, Mexico. *Journal of archaeological science* 148:105692.](http://paperpile.com/b/pxEfRW/hjd8)

[Dierickx, K., S. Presslee, R. Hagan, T. Oueslati, J. Harland, J. Hendy, D. Orton, M. Alexander, and V. L. Harvey. 2022. Peptide Mass Fingerprinting of Preserved Collagen in Archaeological Fish Bones for the Identification of Flatfish in European Waters. *Royal Society open science* 9:220149.](http://paperpile.com/b/pxEfRW/Ebvi)

[Hall, T. A. 2001. BioEdit: A User-Friendly Biological Sequence Alignment Editor and Analysis, Version 5.09. *Department of Microbiology, North Carolina State University, North Carolina*.](http://paperpile.com/b/pxEfRW/qh0O)

[von Holstein, I. C. C., S. P. Ashby, N. L. van Doorn, S. M. Sachs, M. Buckley, M. Meiri, I. Barnes, A. Brundle, and M. J. Collins. 2014. Searching for Scandinavians in Pre-Viking Scotland: Molecular Fingerprinting of Early Medieval Combs. *Journal of archaeological science* 41:1–6.](http://paperpile.com/b/pxEfRW/yy2J)

[Kirby, D. P., M. Buckley, E. Promise, S. A. Trauger, and T. R. Holdcraft. 2013. Identification of Collagen-Based Materials in Cultural Heritage. *The Analyst* 138:4849–4858.](http://paperpile.com/b/pxEfRW/Gsc4)

[Korzow Richter, K., K. McGrath, E. Masson-MacLean, S. Hickinbotham, A. Tedder, K. Britton, Z. Bottomley, K. Dobney, A. Hulme-Beaman, M. Zona, R. Fischer, M. J. Collins, and C. F. Speller. 2020. What’s the Catch? Archaeological Application of Rapid Collagen-Based Species Identification for Pacific Salmon. *Journal of archaeological science* 116:105116.](http://paperpile.com/b/pxEfRW/6GiQ)

[Latch, E. K., J. R. Heffelfinger, J. A. Fike, and O. E. Rhodes Jr. 2009. Species-Wide Phylogeography of North American Mule Deer (Odocoileus Hemionus): Cryptic Glacial Refugia and Postglacial Recolonization. *Molecular ecology* 18:1730–1745.](http://paperpile.com/b/pxEfRW/RPOA)

[McGrath, K., K. Rowsell, C. Gates St-Pierre, A. Tedder, G. Foody, C. Roberts, C. Speller, and M. Collins. 2019. Identifying Archaeological Bone via Non-Destructive ZooMS and the Materiality of Symbolic Expression: Examples from Iroquoian Bone Points. *Scientific reports* 9:11027.](http://paperpile.com/b/pxEfRW/ssy8)

[Rodrigues, A. S. L., A. Charpentier, D. Bernal-Casasola, A. Gardeisen, C. Nores, J. A. Pis Millán, K. McGrath, and C. F. Speller. 2018. Forgotten Mediterranean Calving Grounds of Grey and North Atlantic Right Whales: Evidence from Roman Archaeological Records. *Proceedings. Biological sciences / The Royal Society* 285:20180961.](http://paperpile.com/b/pxEfRW/8vmt)

[Speller, C. F., B. Kooyman, A. T. Rodrigues, E. G. Langemann, R. M. Jobin, and D. Y. Yang. 2014. Assessing Prehistoric Genetic Structure and Diversity of North American Elk (Cervus Elaphus) Populations in Alberta, Canada. *Canadian journal of zoology* 92:285–298.](http://paperpile.com/b/pxEfRW/1lKY)

Strohalm, M., Hassman, M., Košata, B., & Kodíček, M. (2008). mMass data miner: an open source alternative for mass spectrometric data analysis. *Rapid Communications in Mass Spectrometry*, 22(6), 905-908.

[Tamura, K., G. Stecher, and S. Kumar. 2021. MEGA11: Molecular Evolutionary Genetics Analysis Version 11. *Molecular biology and evolution* 38:3022–3027.](http://paperpile.com/b/pxEfRW/XYVz)

[Thompson, J. D., D. G. Higgins, and T. J. Gibson. 1994. CLUSTAL W: Improving the Sensitivity of Progressive Multiple Sequence Alignment through Sequence Weighting, Position-Specific Gap Penalties and Weight Matrix Choice. *Nucleic acids research* 22:4673–4680.](http://paperpile.com/b/pxEfRW/KeFv)

[Vinnikov, K. A., R. C. Thomson, and T. A. Munroe. 2018. Revised Classification of the Righteye Flounders (Teleostei: Pleuronectidae) Based on Multilocus Phylogeny with Complete Taxon Sampling. *Molecular phylogenetics and evolution* 125:147–162.](http://paperpile.com/b/pxEfRW/Dmt4)

[Welker, F., M. Hajdinjak, S. Talamo, K. Jaouen, M. Dannemann, F. David, M. Julien, M. Meyer, J. Kelso, I. Barnes, S. Brace, P. Kamminga, R. Fischer, B. M. Kessler, J. R. Stewart, S. Pääbo, M. J. Collins, and J.-J. Hublin. 2016. Palaeoproteomic Evidence Identifies Archaic Hominins Associated with the Châtelperronian at the Grotte Du Renne. *Proceedings of the National Academy of Sciences of the United States of America* 113:11162–11167.](http://paperpile.com/b/pxEfRW/bqOh)

[Yang, D. Y., B. Eng, J. S. Waye, J. C. Dudar, and S. R. Saunders. 1998. Technical Note: Improved DNA Extraction from Ancient Bones Using Silica-Based Spin Columns. *American journal of physical anthropology* 105:539–543.](http://paperpile.com/b/pxEfRW/srOD)

[Yang, D. Y., and K. Watt. 2005. Contamination Controls When Preparing Archaeological Remains for Ancient DNA Analysis. *Journal of archaeological science* 32:331–336.](http://paperpile.com/b/pxEfRW/hjma)
